# A Systems Biology Approach Reveals the Role of a Novel Methyltransferase in Response to Chemical Stress and Lipid Homeostasis

**DOI:** 10.1371/journal.pgen.1002332

**Published:** 2011-10-20

**Authors:** Elena Lissina, Brian Young, Malene L. Urbanus, Xue Li Guan, Jonathan Lowenson, Shawn Hoon, Anastasia Baryshnikova, Isabelle Riezman, Magali Michaut, Howard Riezman, Leah E. Cowen, Markus R. Wenk, Steven G. Clarke, Guri Giaever, Corey Nislow

**Affiliations:** 1 Department of Molecular Genetics, University of Toronto, Toronto, Canada; 2 Terrence Donnelly Centre for Cellular and Biomolecular Research, University of Toronto, Toronto, Canada; 3 Department of Chemistry and Biochemistry and the Molecular Biology Institute, University of California Los Angeles, Los Angeles, California, United States of America; 4 Banting and Best Department of Medical Research, University of Toronto, Toronto, Canada; 5 Department of Biological Sciences, Yong Loo Lin School of Medicine, National University of Singapore, Singapore, Singapore; 6 Department of Biochemistry, University of Geneva, Geneva, Switzerland; 7 Molecular Engineering Lab, Agency for Science, Technology, and Research, Singapore, Singapore; 8 Department of Pharmacy and Pharmaceutical Sciences, University of Toronto, Toronto, Canada; HudsonAlpha Institute for Biotechnology, United States of America

## Abstract

Using small molecule probes to understand gene function is an attractive approach that allows functional characterization of genes that are dispensable in standard laboratory conditions and provides insight into the mode of action of these compounds. Using chemogenomic assays we previously identified yeast Crg1, an uncharacterized SAM-dependent methyltransferase, as a novel interactor of the protein phosphatase inhibitor cantharidin. In this study we used a combinatorial approach that exploits contemporary high-throughput techniques available in *Saccharomyces cerevisiae* combined with rigorous biological follow-up to characterize the interaction of Crg1 with cantharidin. Biochemical analysis of this enzyme followed by a systematic analysis of the interactome and lipidome of *CRG1* mutants revealed that Crg1, a stress-responsive SAM-dependent methyltransferase, methylates cantharidin *in vitro*. Chemogenomic assays uncovered that lipid-related processes are essential for cantharidin resistance in cells sensitized by deletion of the *CRG1* gene. Lipidome-wide analysis of mutants further showed that cantharidin induces alterations in glycerophospholipid and sphingolipid abundance in a Crg1-dependent manner. We propose that Crg1 is a small molecule methyltransferase important for maintaining lipid homeostasis in response to drug perturbation. This approach demonstrates the value of combining chemical genomics with other systems-based methods for characterizing proteins and elucidating previously unknown mechanisms of action of small molecule inhibitors.

## Author Summary

Chemical genetics uses small molecules to perturb biological systems to study gene function. By analogy with genetic lesions, chemical probes act as fast-acting, reversible, and “tunable” conditional alleles. Furthermore, small molecules can target multiple protein targets and target pathways simultaneously to uncover phenotypes that may be masked by genes encoding partially redundant proteins. Finally, potent chemical probes can be useful starting points for the development of human therapeutics. Here, we used cantharidin, a natural toxin, to uncover otherwise “hidden” phenotypes for a methyltransferase that has resisted characterization. This enzyme, Crg1, has no phenotype in standard conditions but is indispensible for survival in the presence of cantharidin. Using this chemical genetic relationship, we characterized novel functions of Crg1, and by combining diverse genomic assays with small molecule perturbation we characterized the mechanism of cantharidin cytotoxicity. These observations are relevant beyond yeast Crg1 because cantharidin and its analogues have potent anticancer activity, yet its therapeutic use has been limited to topical applications because of its cytotoxicity. Considering that methyltransferases are an extremely abundant and diverse class of cellular proteins, chemical probes such as cantharidin are critical for understanding their cellular roles and defining potential points of therapeutic intervention.

## Introduction

Methyltransferases are a large class of enzymes comprising 0.6–1.6% of protein coding genes in most sequenced organisms [1]. *S*-adenosyl methionine (SAM)-dependent methyltransferases regulate a dynamic network of cellular signaling events and are required to maintain intracellular homeostasis in the face of external perturbations by catalyzing the methylation of a wide variety of substrates (proteins, nucleic acids, lipids and small molecules) [2]–[4]. The characterization and understanding of the roles of most methyltransferases remains challenging, however, due to their dispensability in standard growth conditions. Numerous studies from our lab and others have demonstrated that chemogenomic profiling of the *Saccharomyces cerevisiae* yeast deletion collection [5] is a powerful approach for the identification and subsequent characterization of genes required for growth in the presence of bioactive compounds [6]–[15]. Moreover, while most yeast genes (∼80%) are dispensable for growth in standard laboratory conditions, the presence of chemical and/or environmental perturbations of the cell, 97% of the yeast genome exhibits a fitness defect that would not otherwise have been revealed [15].

Well-established chemogenomic assays in yeast, such as drug-induced Haploinsufficiency Profiling (HIP), Homozygous Profiling (HOP) and Multicopy Suppression Profiling (MSP) are designed to identify small molecule-gene interactions. For example, HIP assay is used to detect compounds that target essential genes, and HOP and MSP are suitable for identification genetic modifiers of drug resistance [8]–[10], [13]. The combination of these chemogenomic assays allowed us to identify a novel gene, *YHR209W*, that we subsequently named *CRG1* (Cantharidin Resistance Gene 1), due to its requirement for growth in the presence of the small molecule cantharidin [14]. Specifically, both *CRG1* heterozygous and homozygous deletion strains exhibited sensitivity to the drug, and the overexpression of *CRG1* conferred resistance to the drug. Nonetheless, Crg1 is uncharacterized, except for annotation derived from large-scale analyses [15]–[17].

Based on its primary sequence, Crg1 is predicted to encode a Class I *S*-adenosyl-methionine (SAM)-dependent methyltransferase [18]. Crg1 shares close sequence homology with *trans*-aconitate methyltransferase Tmt1 (BLAST-P expect value 2×10^−31^ and 3×10^−34^ for the full proteins and the methyltransferase domains, respectively). Tmt1 is known to modify and detoxify small molecules by methylation [19]–[21]. We have previously shown indirectly that Crg1 does not likely possess Tmt1 methyltransferase activity towards *trans*-aconitate, 3-isopropylmalate, and isopropylmaleate, indicating that these closely related proteins have divergent substrates [19], [21]. Bioinformatics analysis from our group has shown, however, that Crg1 clusters with a family of eight methyltransferases based on their methyl-accepting substrate specificity, including Tmt1, the lipid methyltransferases (Coq3, Coq5, and Erg6), and a tRNA methyltransferase, Trm9 [22]. All of these proteins methylate carboxylic acids present in small molecules to form methyl esters, suggesting that Crg1 might have a similar biochemical activity and catalyze the formation of a methyl ester.

Cantharidin, a natural product produced by Chinese blister beetles of the Meloidae family of Coleoptera, is used in Traditional Chinese Medicine for the treatment of a variety of cancers [23]. Cantharidin has potent anticancer activity characterized by cell cycle arrest in G2/M phase, apoptosis, and DNA damage, presumably as a result of the generation of reactive oxygen species [23]–[29], yet its use is limited due to renal and mucous membrane toxicity. Although the activity of cantharidin is usually attributed to its high affinity towards Type 1 and 2A serine/threonine protein phosphatases [30], [31], several studies suggest that cantharidin has additional cellular targets. Specifically, cantharidin has been reported to stimulate xanthine oxidase activity and to inhibit N-acyltransferase and cAMP phosphodiesterase in liver cells, suggesting a complex mode of action [32]–[34]. Using the HIP and HOP genome-wide assays, we discovered that a surprisingly large number of methyltransferase deletion mutants are sensitive to cantharidin, suggesting that, as a class, these enzymes may interact directly or indirectly with cantharidin and participate in the response to cantharidin stress [15]. Notably, among these methyltransferases only the overexpression of *CRG1* is able to confer resistance to cantharidin.

To further explore the function of Crg1 and the mechanism of cantharidin cytotoxicity, we employed chemical genomics tools combined with conventional biological techniques. We demonstrated that Crg1 methylates cantharidin *in vitro*, and identified cantharidin-specific *CRG1* genetic interactors. To extend our chemogenomic results we analyzed the lipid profile of mutants grown in the presence of cantharidin, and demonstrated that cantharidin resistance involves Crg1-dependent maintenance of lipid homeostasis.

## Results

### *CRG1* Is a Functional Methyltransferase Required for Protein Phosphatase Inhibitor Resistance

**Figure 1:**
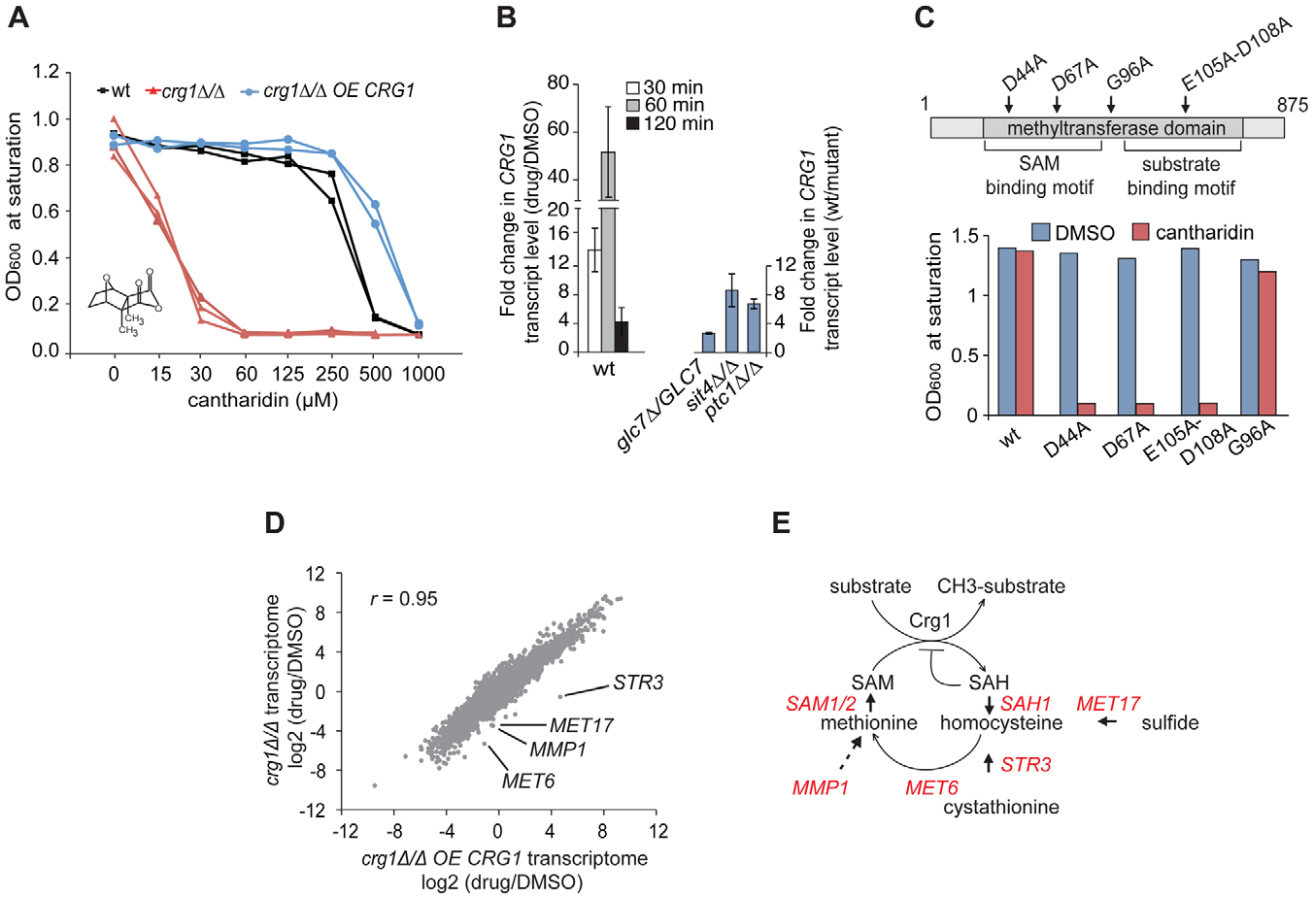
Functional SAM-dependent methyltransferase Crg1 is required for cantharidin response. (A) *CRG1* gene dose is important for cantharidin tolerance. Wt, *crg1Δ/Δ* and *CRG1*-overexpressing *crg1Δ/Δ* mutants were assessed in the presence of cantharidin in YPD. Dose-response curves were obtained by plotting OD_600_ at saturation point *vs.* tested drug concentrations. (B) Chemical (left panel) and genetic inhibition (right panel) of protein phosphatases result in the induction of *CRG1*. Wt, *glc7Δ/GLC7* heterozygous, *sit4Δ/Δ* and *ptc1Δ/Δ* homozygous deletion mutants grown to mid-exponential phase were incubated with or without cantharidin (250 µM). For each time point, total RNA was extracted, cDNA synthesized and the relative abundance of *CRG1* transcript was analyzed by qRT-PCR. Data are the mean of at least three independent experimental replicates, and error bars represent the standard deviation. (C) Point mutations in conserved residues of the Crg1 methyltransferase domain reduce cantharidin tolerance. Site-specific mutations in the conserved motifs of methyltransferase domain are shown with the arrows. Mutated *CRG1* ORFs were cloned under *GAL1* promoter and transformed into *crg1Δ/Δ* cells. The transformants were grown in SD-URA with raffinose (2%) to mid-exponential phase and induced with galactose (2%). The fitness of point mutants based on saturation at final OD was assessed in the presence of cantharidin (6 µM). (D) A plot comparing the transcriptome profiles of *crg1Δ/Δ* and *CRG1*-overexpressing *crg1Δ/Δ* mutants. Exponentially grown cells were treated with cantharidin (250 µM) for 1 hour or DMSO, total RNA was extracted and synthesized cDNA was hybridized to Affymetrix Tiling arrays. Significantly different GO Biological processes are listed in Table S1. (E) Diagram showing that the methionine biosynthesis is tightly linked to SAM cycling in methylation reactions coordinated by methyltransferases. The genes that are transcriptionally different between cantharidin-resistant and sensitive mutant are shown in red. *STR3* (*P*-value <0.0057), *MET17* (*P*-value <0.0036), *MET6* (*P*-value <0.012), *SAM1* (*P*-value <0.02), *SAH1* (*P*-value <0.03), *MMP1* (*P*-value <0.04), *SAM2* (*P*-value <0.056) (Table S2).

To confirm our published cantharidin-specific response of *CRG1* [14], we measured the growth of three strains 1) wild-type diploid strain BY4743, 2) a *crg1 Δ/Δ* homozygous deletion strain, and 3) a *crg1Δ/Δ* homozygous deletion strain overexpressing *CRG1* (2 µ plasmid) as a function of cantharidin concentration. We observed that the gene dosage of the putative SAM-dependent methyltransferase *CRG1* correlated with the sensitivity/resistance of these strains to cantharidin (Figure 1A). In agreement with this gene-dose dependent effect, *crg1Δ/CRG1* heterozygous mutants grew worse than the wild-type strain but better than a *crg1Δ/Δ* homozygous mutant in the presence of cantharidin (500 µM) (Figure S1A). We found that cantharidin is more potent against cells grown in synthetically defined (SD) medium than in YPD medium (5 µM and 250 µM, IC_20_ for wild-type in SD and YPD, respectively; Figure S1B). The observed differential drug sensitivity in defined media and rich YPD media is a common phenomenon in our drug screens (unpublished data). We also tested structural analogues of cantharidin, including cantharidic acid and norcantharidin, and found that these compounds produced a similar gene-dose dependent response in *crg1* mutants (Figure S1C).

Because our data suggested that *CRG1* responds to cantharidin in a gene dose-dependent manner, we next tested whether the transcription of *CRG1* is induced in the presence of the drug. qRT-PCR analysis showed that the relative abundance of *CRG1* transcripts increased drastically in the wild-type strain after 60 min of the drug treatment (250 µM) compared to the DMSO control (*P*-value <0.02; Figure 1B, left panel). Importantly, a gene-dose dependent effect in the response to cantharidin was also observed for *CRG1* transcript levels in *crg1Δ/CRG1* heterozygous and *CRG1*-overexpressing mutants (Figure S2A). In agreement with the qRT-PCR data, we also observed induction of Crg1 at the protein level. GFP-tagged Crg1 protein increased from undetectable levels prior to treatment and accumulated to high levels (restricted to the cytoplasm) following 1 hour of cantharidin treatment (Figure S1B).

Given the known high affinity of cantharidin towards Type 2A protein phosphatases (PP2A) and to a lesser degree towards Type 1 (PP1) [30], [31], we tested if *CRG1* induction was mediated by chemical inhibition of protein phosphatase function. We phenocopied cantharidin treatment using a panel of protein phosphatase homozygous deletion strains. Consistent with the results of chemical inhibition of protein phosphatases with cantharidin, we found that the homozygous deletion strains *sit4Δ/Δ* (PP2A), *ptc1Δ/Δ* (PP2C) and the heterozygous deletion strain *glc7Δ/GLC7* (PP1) also resulted in transcriptional upregulation of *CRG1* in the absence of cantharidin (Figure 1B, right panel). It is important to note that perturbation of these protein phosphatases accounted for only ∼20% of the transcript induction observed by cantharidin. Furthermore, the treatment with calyculin A, a structurally distinct PP2 and PP1 inhibitor [35], known to interact with the yeast PP1 *GLC7* [14], resulted in an increase of *CRG1* transcript level to a similar degree as in *glc7Δ/GLC7* mutant (∼2.5 fold; Figure S2C). This observation opens up the possibility that cantharidin acts independently of this PPase. This hypothesis is also supported by our observation that overexpression of *GLC7* confers resistance to calyculin A, but not to cantharidin [14]. These results also suggest that these protein phosphatases are likely to be negative regulators of the cellular pathway regulating *CRG1* induction.

We also observed that cantharidin-induced transcription of *CRG1* follows a temporal pattern characteristic of diverse environmental stress responses [36], following a peak at 60 min of treatment the transcript levels began to decrease at 120 min (∼40 fold, *P*-value<0.01; Figure 1B). Indeed, a comprehensive genome-wide analysis of diverse environmental stresses from publicly available expression data [36] revealed that the transcription profile of *CRG1* in diverse stress conditions correlates highly (*r* = 0.8) with a well-characterized stress-responsive gene, the heat shock protein *SSE2* (Figure S2D), suggesting that *CRG1* is also transcriptionally activated by other stress conditions in addition to cantharidin.

Because *CRG1* is annotated (based on its amino acid sequence) as a putative SAM-dependent methyltransferase [18], we next asked whether its methyltransferase domain is required for cantharidin tolerance by mutating amino acids (D44A, D67A, E105A-D108A) within the conserved motifs (Figure 1C). These amino acids have previously been shown to be critical for activity of other methyltransferases [37]. Overexpression of these *crg1* site-specific mutants in a *crg1Δ/Δ* strain failed to confer cantharidin resistance while, in contrast, mutation of a non-conserved residue (G96A) in the methyltransferase domain showed resistance equivalent to wild-type *CRG1* (Figure 1C). The observed decrease in resistance to cantharidin was not due to reduced expression of the mutated Crg1 proteins (Figure S2E), suggesting that the methyltransferase domain of Crg1 is both functional and important for cellular survival in the presence of the drug.

To identify other potential cellular factors important for Crg1-mediated cantharidin resistance, we profiled the complete yeast transcriptome using whole-genome tiling microarrays. Transcriptional changes in wild type, *crg1Δ/Δ* deletion and *CRG1*-overexpressing *crg1Δ/Δ* strains were analyzed after 1 hour of exposure to the drug. To ensure that the transcriptome datasets for the different strains are comparable, the IC_20_ for wild type (250 µM) was applied to all strains. Even at this high dose, the hypersensitive *crg1Δ/Δ* strain is viable, after 1 hour of exposure (Figure S3A). When applied for an extended period, this dose is, in fact, inhibitory for growth of *crg1Δ/Δ* strains (Figure 1A and Figure S1A). Furthermore, the treatment of *crg1Δ/Δ* strain with a lower dose (30 µM, the IC_20_ for this mutant) resulted in quantitative difference in the transcriptome profile rather than in any qualitative differences, suggesting that the transcriptional changes are consistent across a range of concentrations. In particular, this observation was relevant to downregulated genes (Figure S3B and S3C). It is also worth noting that the expression of most genes was not affected by *crg1* mutation. To uncover cantharidin-specific genes in our transcriptome analysis, we eliminated Environmental Stress Response (ESR) genes known to be activated by a large number of stresses, such as genes required for vacuole biogenesis, response to stress, ribosome biogenesis, and RNA processing [36]. We also eliminated those genes that did not demonstrate at least two-fold difference in the presence of cantharidin or if their differential expression failed to show statistical significance. To detect genes and biological processes that are differentially expressed among the strains and treatments, the enrichment of genes for Gene Ontology (GO) term Biological process in the transcriptomes of wild type, *crg1Δ/Δ* and *CRG1*-overexpressing *crg1Δ/Δ* strains in the presence and absence of cantharidin were compared (Table S1). We also clustered genes according to their expression pattern. The clustering and GO term comparative analysis revealed that significantly downregulated genes (log2 (drug/DMSO) <−1, *P*-value <0.05) in *crg1Δ/Δ* mutant and the wild type were enriched for the genes of amino acid process (multiple-testing corrected *P*-value <1.0×10^−7^ and *P*-value <7.0×10^−7^, respectively), while the transcriptional profile of cantharidin-resistant *CRG1*-overexpressing *crg1Δ/Δ* strain did not demonstrate a similar enrichment (Figure 1D and Figure S3D). Of particular interest, most of the genes that comprise methionine biosynthetic process (*MET6*, *MET17*, *MMP1*, *STR3*, *ADE3*, *SAM1*, *SAM2*, *SAH1*, *MET22*, *MET31*) were differentially expressed between cantharidin-resistant *CRG1*-overexpressing *crg1Δ/Δ*, wild type and cantharidin-sensitive *crg1Δ/Δ* strain in the presence of cantharidin (*P*-value <0.05; Table S2; Figure 1E). One noteworthy example is *STR3*, a cystathionine beta-lyase, the gene that demonstrated the most differential expression in the strains. *STR3* was significantly induced by cantharidin in *CRG1*-overexpressing *crg1Δ/Δ* strain (log2 (drug/DMSO) = 4.7, *P*-value <0.022) and downregulated in the wild type (log2 = −0.6) and *crg1Δ/Δ* (log2 = −0.55). The observed differential expression of *STR3* was further confirmed by qRT-PCR (Figure S3F). Str3 is of interest because it functions in methionine biosynthesis by converting cystathionine into homocysteine, a precursor for methionine, which is a substrate for the generation of SAM. SAM is required as a methyl donor for methylation reactions (Figure 1E). To further explore the role of the Crg1 SAM-dependent methyltransferase, we treated wild-type cells with a combination of cantharidin and *S*-adenosyl homocysteine (SAH), a non-specific methyltransferase inhibitor. We found that wild-type strains were more sensitive to the cantharidin/SAH combination compared to either single agent (Figure S3F). These observations confirm the requirement of SAM-dependent methyltransferase activity in response to cantharidin, and suggest that Crg1 is a functional methyltransferase that catalyzes a SAM-dependent methylation reaction important for cantharidin resistance.

### Crg1 Methylates Cantharidin *In Vitro*

**Figure 2:**
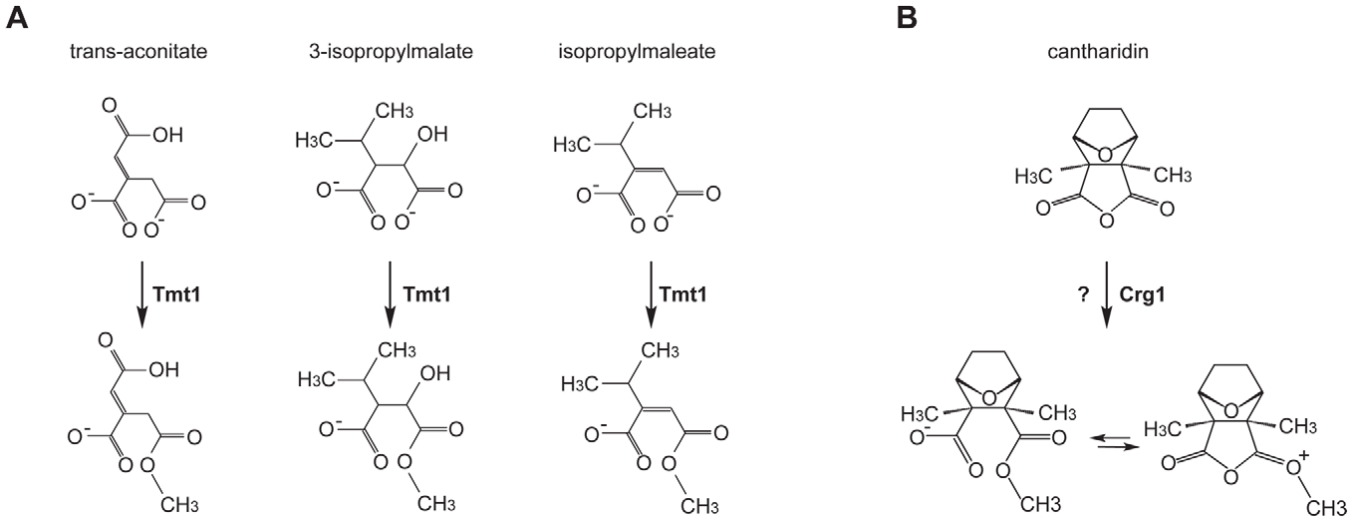
Small molecule methyltransferase TMT1 is a sequence homolog of Crg1. (A) Tmt1, a sequence homolog of Crg1, modifies small molecule intermediates of TCA cycle (*trans*-aconitate) and leucine biosynthesis (3-isopropylmalate and isopropylmaleate) to form methyl esters. (B) Hypothetical methylation of cantharidin by Crg1.

**Figure 3:**
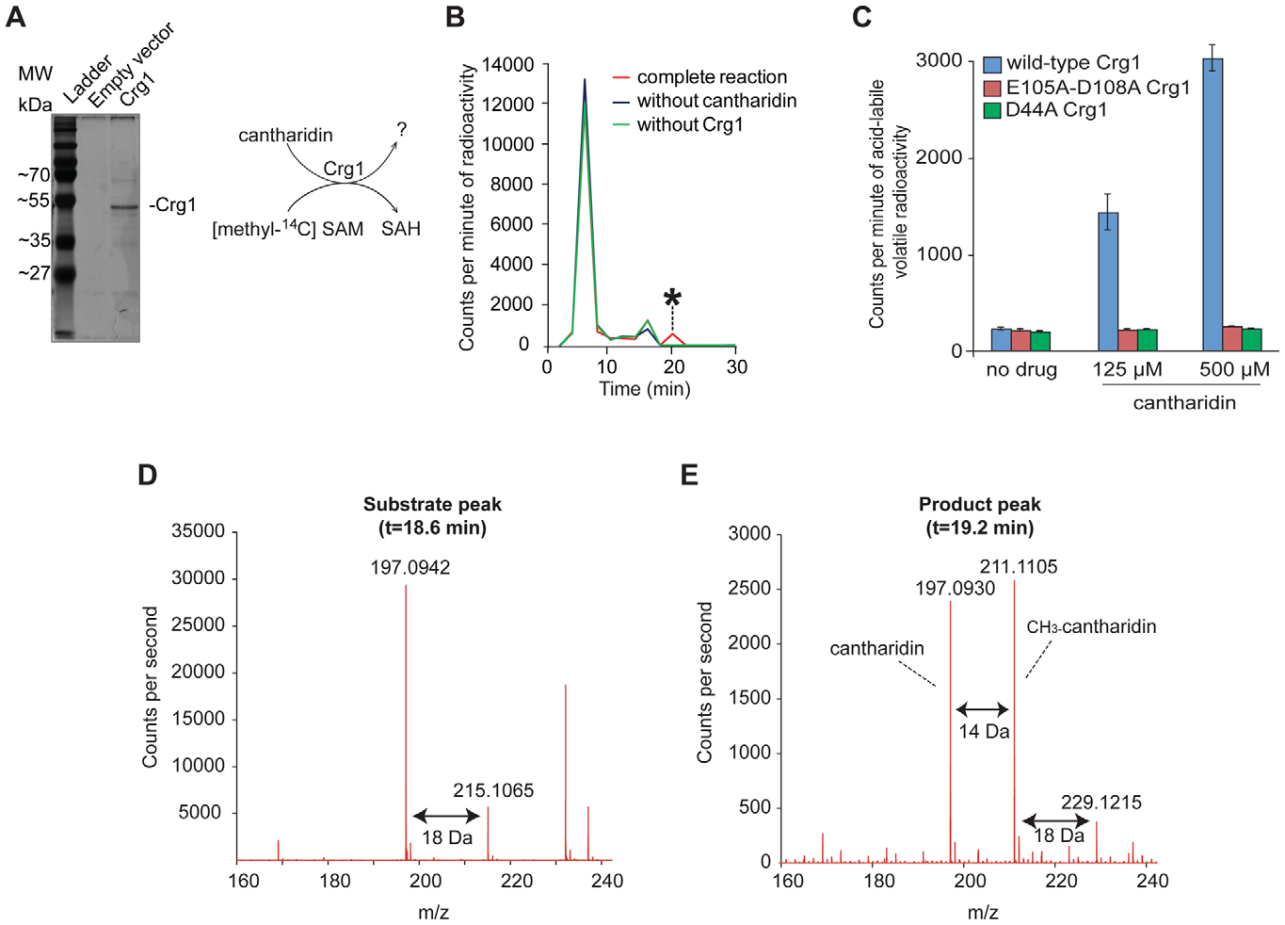
Crg1 methylates cantharidin *in vitro*. (A) Silver stained 12% SDS-PAGE of purified His-tagged Crg1. Wild-type cells (Y258) carrying empty vector *BG1805* and *BG1805-GAL1-CRG1* were grown in SD-URA and raffinose (2%) to mid-exponential phase. The expression of *CRG1* was induced with galactose (2%) for 5 hours. His-tagged Crg1 was purified with Ni-sepharose resin. The diagram (right panel) shows the transfer of [*methyl*-^14^C] from *S*-adenosyl-[*methyl*-^14^C]methionine to a potential substrate by purified Crg1. (B) *In vitro* enzymatic reaction mixtures containing cantharidin, *S*-adenosyl-[*methyl*-^14^C]methionine, and Crg1 were separated by reverse phase chromatography. Radioactivity in the fractions was quantified with a scintillation counter, and a cantharidin and Crg1-dependent peak with a retention time of 18–20 minutes was identified (asterisk). (C) *In vitro* analysis of the reactions containing cantharidin and mutated forms of Crg1 by measurement of the amount of acid-labile volatile radioactivity (see Materials and Methods for details). (D) Additional *in vitro* reaction with unlabeled SAM were prepared in a similar manner and analyzed by liquid chromatography-mass spectrometry with positive ionization. The mass spectrum of the peak from the full reaction with an elution time of 18.6–18.8 minutes is shown. (E) The mass spectrum of the peak with an elution time of 19.2 minutes from the complete reaction with cantharidin, SAM and Crg1.

Given that *CRG1* provides cantharidin resistance in a gene dose-dependent manner and because of its close sequence homology to *TMT1*, a small molecule methyltransferase that catalyzes the formation of methyl esters (Figure 2A), we hypothesized that Crg1 might methylate cantharidin because this drug bears some structural similarity to the substrates of Tmt1 (Figure 2B). To test this possibility, we performed *in vitro* biochemical assays with purified Crg1, cantharidin, and *S*-adenosyl-[*methyl*-^14^C]methionine (Figure 3A). These *in vitro* reactions were separated via reverse phase liquid chromatography and the radioactivity of the collected fractions was quantified with a scintillation counter. We detected a unique peak of radioactivity eluting in the 18–20 min fraction (Figure 3B). The appearance of this peak was both cantharidin and Crg1-dependent, suggesting that it could correspond to methylated cantharidin.

To confirm that the novel activity was catalyzed by Crg1 rather than by a co-purifying protein, we repeated the methylation reactions with mutant forms of Crg1 containing amino acid substitutions at critical residues within the methyltransferase domain. As described earlier, the D44A and E105A-D108A mutations abolished resistance to cantharidin (Figure 1C), so we assessed whether these mutated proteins were able to methylate the drug molecule. We prepared *in vitro* reactions containing varying concentrations of cantharidin and quantified the amount of acid-labile volatile radioactivity because methyl esters are known to readily hydrolyze in both strongly acidic and basic conditions to yield methanol [19], [38]. Unlike the reactions performed with wild-type Crg1, addition of cantharidin to the reactions with mutant forms of the enzyme showed no increase in acid-labile radioactivity (Figure 3C), strongly suggesting that a functional methyltransferase domain in Crg1 is required for cantharidin methylation.

To definitively determine whether cantharidin is a substrate of Crg1, we prepared and analyzed unlabeled reactions containing purified Crg1, cantharidin, and SAM by liquid chromatography-mass spectrometry. We first looked at the extracted ion chromatogram expected for unreacted cantharidin (C_10_H_13_O_4_^+^; m/z = 197.0814±100 ppm) and found a large peak in cantharidin-containing reactions with an elution time of 18.6–18.8 min (Figure S4B). The combined spectra of this peak in the complete reaction mixture contained several species: m/z = 197.0942 and 215.1065, corresponding to the m/z for cantharidin and hydrated cantharidin (C_10_H_15_O_5_^+^; m/z = 215.0919), respectively (Figure 3D). Because these species co-elute at 18.6–18.8 min in the complete reaction and the control reactions containing cantharidin (Figure S4B and S4C) and because sterically hindered anhydrides do not favor hydrolysis [39], a likely explanation is that cantharidin forms a co-eluting water adduct during ionization.

Next, we analyzed the full reaction to determine whether Crg1 methylates cantharidin. Specifically, we analyzed the extracted ion chromatogram expected for methyl cantharidin (C_11_H_15_O_4_^+^; m/z = 211.0970±100 ppm). We identified a peak eluting after cantharidin at 19.2 min corresponding to the mass of methyl cantharidin in the complete reaction mixture (Figure S4D). Importantly, this species was absent in each of our control reactions lacking cantharidin, SAM, or Crg1 (Figure S4D). This is strong evidence that cantharidin is indeed methylated by Crg1. Finally we analyzed the combined spectra of the 19.2-min peak (Figure 3E). In addition to the m/z = 211.1105 species, we observed m/z = 229.1215 and m/z = 197.0930 species, corresponding to hydrated methyl cantharidin (C_11_H_17_O_5_^+^; m/z = 229.1076) and cantharidin, respectively. Importantly, all of these species co-elute (Figure S4B, S4D and S4E).

Based on its close sequence homology to Tmt1, we suspect that Crg1 catalyzes the formation of a cantharidin methyl ester (Figure 2). In solution, this putative methyl ester is likely in equilibrium with its ring-closed methyl anhydride form. If the equilibrium favors the ester form, some fraction of the cantharidin methyl ester could undergo ring-closing elimination reactions during ionization to yield methyl cantharidin and cantharidin products. Likewise, if the equilibrium favors the methyl anhydride, it may possibly undergo in-source fragmentation to give cantharidin, and like cantharidin, it may simply form a water adduct during ionization. Although our mass spectrometry data strongly support our hypothesis that Crg1 methylates cantharidin *in vitro*, additional analysis is needed to determine the structure of the methylated drug molecule. It is possible that the observed methyl cantharidin and hydrated methyl cantharidin species are ionization products of another methylated cantharidin derivative that is the actual product of the Crg1-catalyzed reaction.

### Revealing the Genetic Interactome of *CRG1* in the Presence of Cantharidin

**Figure 4:**
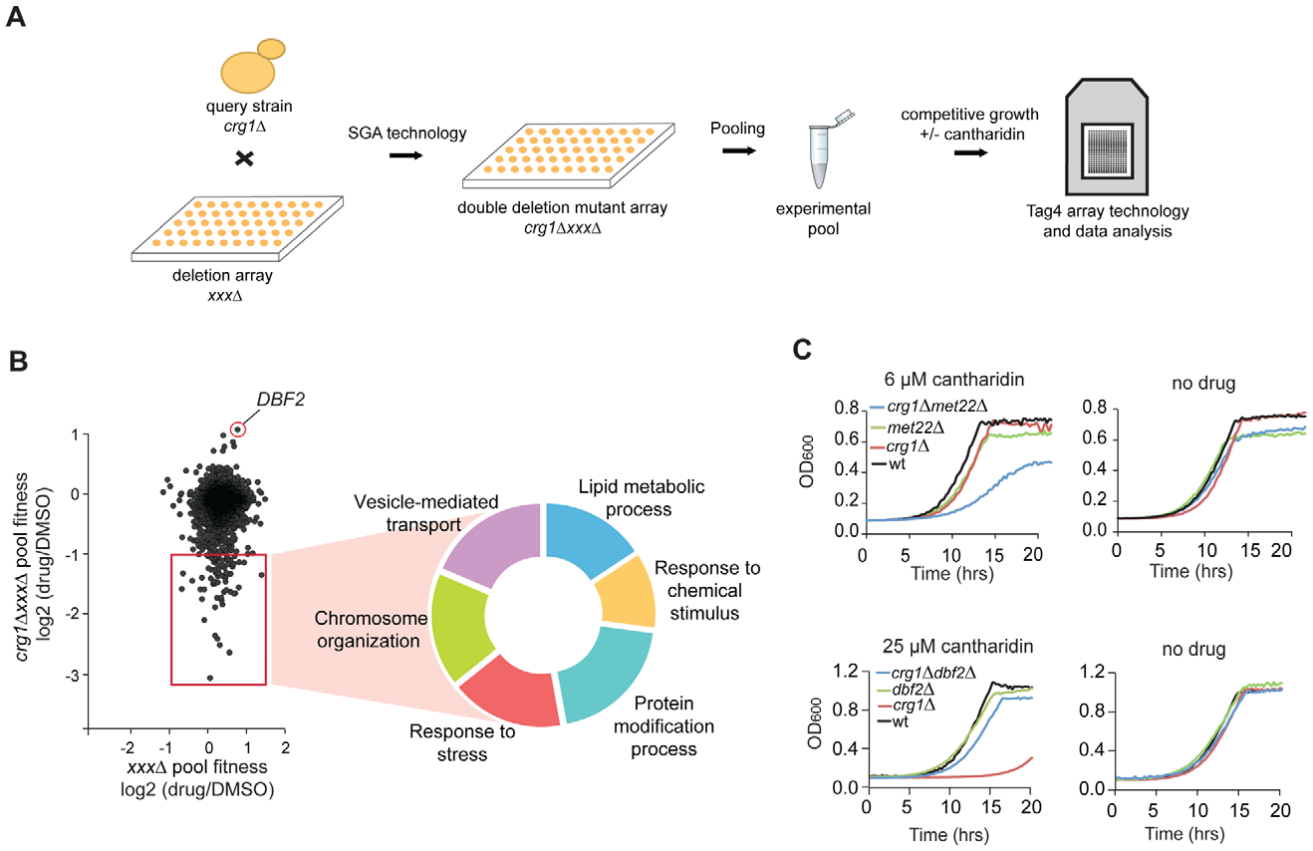
Characterization of cantharidin-specific genetic interactome of *CRG1*. (A) Experimental scheme for analysis of cantharidin-specific genetic interactors of *CRG1*. Double deletion mutants *crg1ΔxxxΔ* generated through Synthetic Genetic Array (SGA) were pooled together and treated with cantharidin (30 µM) for 20 generations in YPD. Genomic DNA was isolated, unique strain-representative barcodes were PCR amplified, and the PCR products were hybridized to TAG4 arrays for the quantitative analysis of fitness of the mutants (see Materials and Methods for details). (B) Scatter plot representing cantharidin-gene interactions obtained from the comparative analysis of *ura3ΔxxxΔ* (control single deletion pool) and *crg1ΔxxxΔ* pools. *CRG1*-dependent interactors are highlighted in the red square. The hits are obtained from the averaged datasets (n = 6 for *crg1ΔxxxΔ* pools and n = 4 for *ura3ΔxxxΔ*). Significant negative genetic interactors were categorized according to their biological processes (*P*-value <0.002 before multiple testing correction) (Table S3). (C) Representative growth curves for the top hits (sensitive and resistant) that genetically interact with *CRG1* in the presence of cantharidin. Cells were grown in YPD media with and without cantharidin. *met22* and *dbf2* deletion strains were treated with cantharidin to test their sensitivity and resistance, respectively.

To identify cellular processes required for cantharidin resistance and to define the spectrum of genes that compensate for the absence of *CRG1* in the presence of cantharidin, we used a chemogenomic approach to analyze genetic interactions between *CRG1* and each of the ∼4800 non-essential yeast genes both in the presence and absence of the drug. A genetic interaction between two genes occurs when the phenotype of the double deletion mutant shows significant deviation in fitness compared with the expected (multiplicative) effect of combining two single mutants (e.g. sickness or synthetic lethality) [17], [40]. When synthetic lethality is observed, it suggests that the genes may have overlapping functions. By analogy, identification of drug-gene interactions will similarly uncover genes that act in parallel with a gene of interest and these interactions can illuminate a compound's effect on a cell. To perform this experiment, we generated double deletion mutants (with a *crg1Δ* strain as the query) using Synthetic Genetic Array (SGA) technology [40], pooled all viable double deletion mutants and analyzed their growth in a competitive fitness assay in the presence and absence of cantharidin (Figure 4A) [41]. Six highly reproducible and independent *crg1ΔxxxΔ* pools (*r* = 0.72, Figure S5A) were further averaged yielding 70 double deletion mutants (Table S3; Datasets S1 and S2) that showed significant growth defects (log2 (drug/DMSO) <−1, *P*-value <0.05) in the presence of an IC_20_ dose of cantharidin (30 µM) in YPD when grown in a pool. Noteworthy, these genes were not sensitive as single deletion mutants (*P*-value <0.025; Figure 4B and 4C, Table S3), and, thus, the effect was specific to the double mutant combination. To obtain a general overview of “aggravating” (negative) interactors of *CRG1* in the presence of cantharidin, we categorized this set of genes according to their GO term Biological process (Figure 4B). This dataset comprised diverse biological processes, including vesicle-mediated transport (*P*-value <0.008), chromosome organization (*P*-value <0.001), response to chemical stimulus (*P*-value <0.019), lipid metabolic process (*P*-value <2.0×10^−5^), response to stress (*P*-value <0.018), and protein modification process (*P*-value <0.003).

We also identified the serine/threonine kinase *DBF2* as a strong suppressor of *CRG1*-dependent cantharidin toxicity (log2 (drug/DMSO) = 1.12, *P*-value <4.0×10^−5^; Figure 4B). This interaction was confirmed by evaluating the fitness of individual strains in liquid and on solid SD medium in the presence of 25 µM and 10 µM cantharidin, respectively (lethal doses for *crg1Δ* strain in these media conditions; Figure 4C and Figure S5B). Furthermore, the alleviating interaction between *DBF2* and *CRG1* was not observed at 37°C, indicating cantharidin-specific nature of this interaction (Figure S5B). In addition to the well-characterized roles of Dbf2 in the mitotic exit network [42], this newly uncovered interaction suggests that this kinase may have an opposing function to the protein phosphatases (PP2A and PP1), the primary targets of cantharidin [30], [31]. As independent evidence for an interaction with cantharidin, we found that *DBF2* transcript levels were significantly decreased in a *crg1Δ/Δ* mutant in the presence of cantharidin (250 µM) (∼2-fold, *P*-value <0.013) compared to DMSO control, and that change was not detected in other strains (Figure S5C). This observation confirms the role of Crg1 in phosphorylation/dephosphorylation homeostasis during cantharidin stress.

### *CRG1* Is Important for Lipid Homeostasis during Cantharidin Treatment

**Figure 5:**
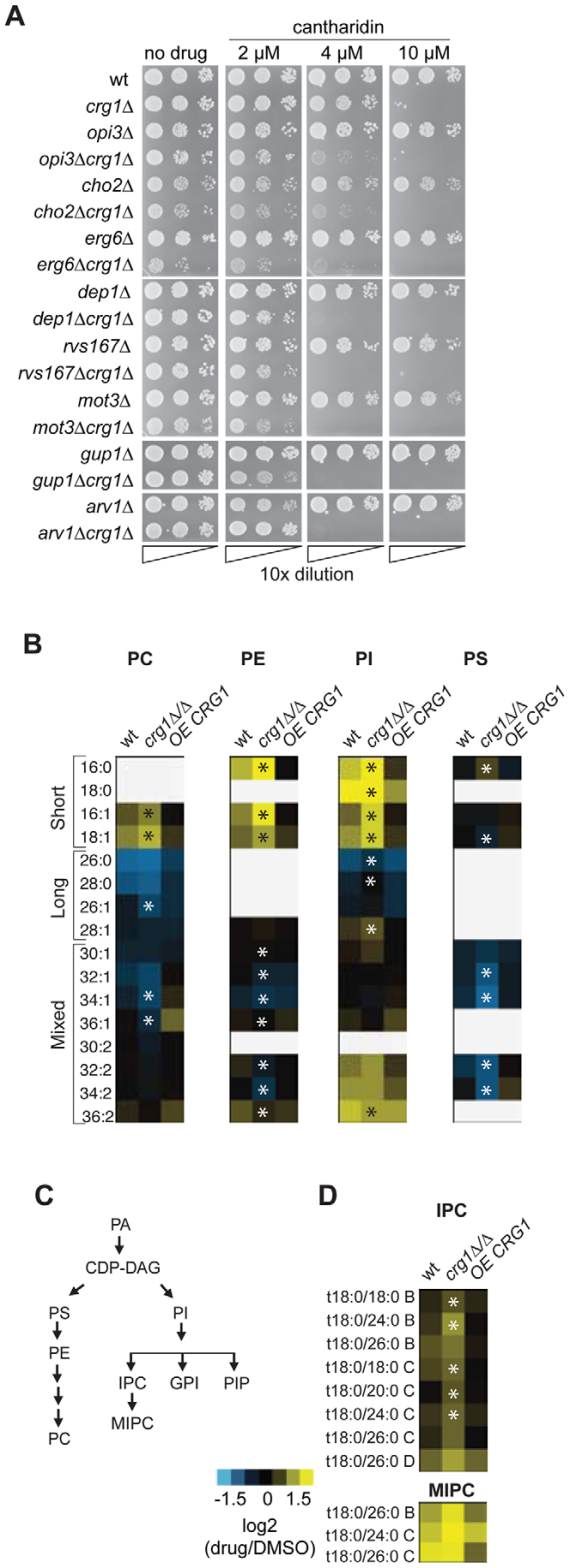
Crg1 is important for lipid homeostasis during cantharidin stress. (A) *CRG1* is synthetically lethal with lipid-related genes in the presence of cantharidin. Cells were normalized to an equivalent OD_600_,10-fold diluted, spotted onto synthetic complete defined medium containing cantharidin and incubated at 30°C. (B) Comparative phospholipid profiles of wild type and *crg1* mutants in response to cantharidin. Cells grown to mid-exponential phase in YPD were treated with cantharidin (250 µM) for 2 hours. Lipid standards were added to the cells, and extracted lipids were measured using ESI-MS/MS. The quantities of lipid species are expressed as ion intensities relative to the levels in DMSO, and converted to a log2 scale. Data are the average of three samples. Statistical significance in the abundance of lipid species in the presence of cantharidin between wild type and mutants was determined using Kruskal-Wallis test, *P-value <0.05 (Table S4). (C) A simplified diagram of phospholipid biosynthesis linked to sphingolipid biosynthesis. PIs species contribute to biosynthesis of complex sphingolipids, GPI anchors, and PIPs. (D) Comparison of sphingolipid profiles of wt, *crg1Δ/Δ* and *CRG1*-overexpressing *crg1Δ/Δ* mutants in the presence of cantharidin (250 µM). The suffixes -B, -C, and -D on IPC and MIPC denote hydroxylation states, having two, three, or four hydroxyl groups, respectively. Statistical significance in the abundance of lipid species in the presence of cantharidin between wild type and mutants was determined using Kruskal-Wallis test, *P-value <0.05 (Table S4).

To identify genes specifically required for growth in the presence of cantharidin, we removed the genes that behave as multidrug resistance genes (MDR) from our chemogenomic dataset described above. MDR genes are defined here as those that are required for growth in the presence of multiple stress conditions (at least 20% of tested conditions for homozygous deletion strains) [15]. This filtering removed apparent enrichment of genes involved in vesicle-mediated transport genes (*P*-value = 0.216), response to stress (*P*-value = 0.024), chromosome organization (*P*-value = 0.075) and protein modification process (*P*-value = 0.021). Following this, we found that cantharidin-specific *CRG1* interactors are significantly enriched for genes required for lipid metabolic process (multiple testing corrected *P*-value <0.0003) (Figure S6A). In particular, lipid methyltransferases (*CHO2*, *ERG6*), glycosylphosphatidylinositol (GPI) lipid biosynthesis genes (*ARV1*, *GUP1*, *PER1*) and lipid-related genes (*SAC1*, *MOT3*, *DEP1*, *RVS167*, *YTA7*) are essential in the double deletion strains in the presence but not absence of cantharidin treatment (Figure 5A). Furthermore, we demonstrated that an increase in cantharidin concentration (10 µM) did not result in cantharidin sensitivity for these genes compared to wild type (Figure 5A), confirming the dependence of the detected interactions on the presence of *CRG1*.

To explore further the role of *CRG1* in lipid metabolism and related processes, we compared the lipid content (or “lipidome”) of wild type, *crg1Δ/Δ* homozygous deletion and *CRG1*-overexpressing *crg1Δ/Δ* strains in the presence and absence of cantharidin (250 µM) using electrospray ionization tandem mass spectrometry (ESI-MS/MS) analysis [43]. We observed significant changes in the abundance of most glycerophospholipids and sphingolipids in both the wild type and *crg1Δ/Δ* strains after growth in cantharidin-containing medium (*P*-value <0.05, Kruskal-Wallis test). The strains with a *CRG1*-overexpressing construct did not exhibit significant cantharidin-induced lipid alterations (*P*-value >0.08, Kruskal-Wallis test; Figure 5B, Table S4, Dataset S3). Specifically, in both the wild type and *crg1Δ/Δ* strains, cantharidin measurably increased the levels of short chain phosphatidylcholine (PC), phosphatidylethanolamine (PE), and phosphatidylinositol (PI) species, while the levels of long-chain PCs and PIs were reduced (Figure 5B). In the *crg1Δ/Δ* strain we also noted a substantial decrease in the levels of mixed size phosphatidylserine (PS) species after cantharidin stress, while the wild type and *crg1Δ/Δ* strain had increased levels of saturated short chain (C16 and C18) PI species compared to mono-unsaturated short chain PIs in cantharidin (Table S4). Such abundance changes with respect to acyl chain length and saturation were not observed in the *CRG1*-overexpressing mutant, suggesting that extra copies of *CRG1* complemented the cantharidin-induced defects.

It has been previously reported that phospholipid and sphingolipid biosynthetic pathways are interconnected (Figure 5C) [44]–[46]. One way in which this interconnection is seen is when, a single gene deletion or chemical perturbation of cells results in the so-called “ripple effect” [45] characterized by lipidome-wide perturbations. We see evidence of this effect: the amounts of the most abundant sphingolipid inositolphosphoceramide (IPC) and mannosyl-inositolphosphoceramide (MIPC) were also affected by cantharidin in a *crg1Δ/Δ* mutant (Figure 5D). To investigate if other lipid intermediates are affected by the drug in a similar manner in *crg1* mutants, we analyzed both sterol content and the formation of lipid droplets, which serve as storage pools of triacylglycerols and steryl esters [47]. We found no obvious changes in these lipid species in the presence of drug (Figure S6B and S6C). Taken together, these results demonstrated that cantharidin's effect is specific towards phospholipids and sphingolipids in *crg1* mutants.

To test if cantharidin-induced alterations in yeast lipidomes are evolutionally conserved, we examined the lipidome of the human fungal pathogen *Candida albicans* in response to cantharidin. A *C. albicans* homozygous *crg1* deletion (*orf19.633Δ/Δ*) displayed similar growth defects to those observed in *S. cerevisiae* when challenged with cantharidin (Figure S7A). Lipidomic analysis demonstrated that cantharidin treatment (2 mM, IC_20_ for *C. albicans* wild type) resulted in the significant changes in most phospholipid species in both wild type and *orf19.633Δ/Δ* homozygous mutant (*P*-value <0.05). Furthermore, although to a more modest degree than seen in *S. cerevisiae*, we found that *C. albicans CRG1* may account for some difference between wild type and a mutant strain (*P*-value <0.05; Figure S7B; Dataset S4). In addition, we have shown previously that the overexpression of *C. albicans* ORF *orf19.633* restored cantharidin resistance in *S. cerevisiae crg1Δ/Δ* mutant [14], further suggesting that the lipid homeostasis functions of this *C. albicans* putative SAM-dependent methyltransferase are conserved.

### Crg1-Dependent Effects of Cantharidin on Cytoskeleton Organization

**Figure 6:**
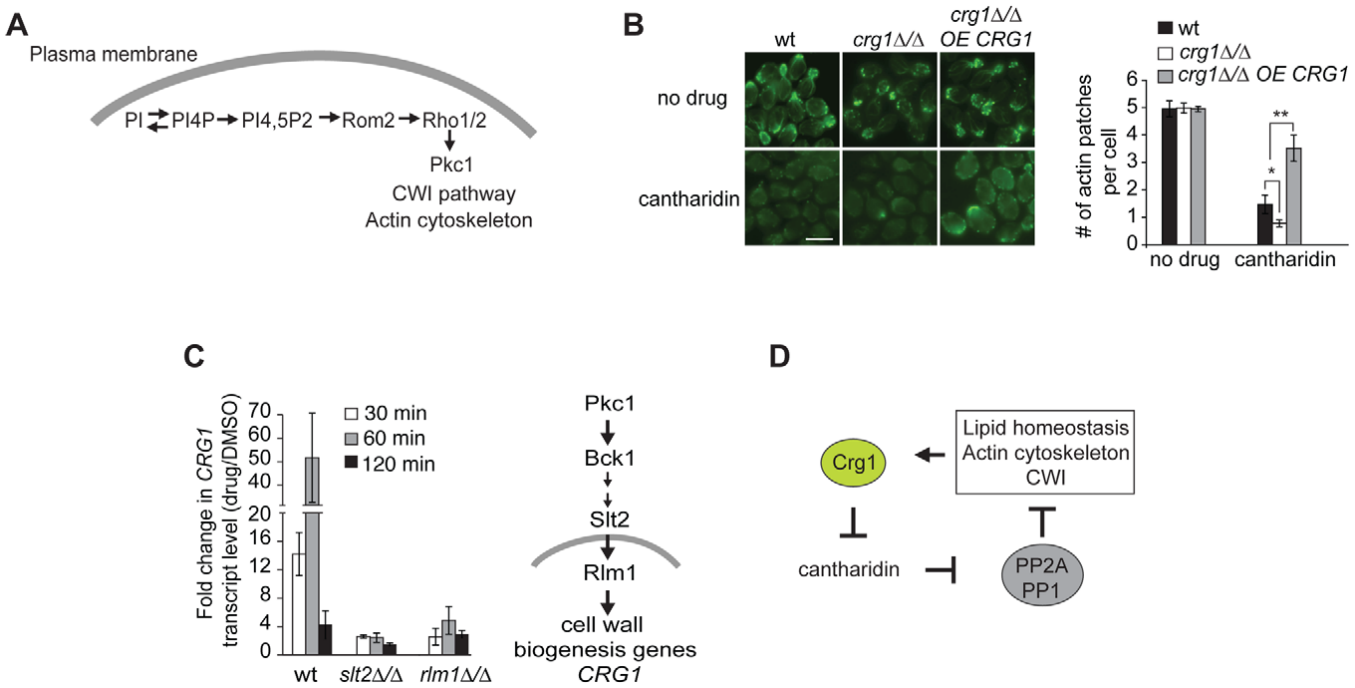
Crg1 is important for actin patch formation during cantharidin treatment. (A) Diagram showing how PIP species are involved in Pkc1-dependent changes in actin cytoskeleton. (B) Crg1 is important for actin patch integrity during cantharidin stress. Wt, *crg1Δ/Δ* mutant and *CRG1*-overexpressing *crg1Δ/Δ* cells were grown to mid-exponential phase at 30°C in YPD in the presence of cantharidin (250 µM) or DMSO for 1 hour. Cells fixed with formaldehyde were stained for actin with FITC-phalloidin and visualized by fluorescence microscopy. Bar, 5 µm. The number of actin patches per cell in each sample was quantified. Values are the mean of three independent replicates (n = 270–1000), error bars are the standard deviation; * *P*-value <0.025, ** *P*-value <0.0002 (Student's t-test). (C) Cantharidin induces *CRG1* transcription via the Cell Wall Integrity (CWI) pathway. Cells grown to mid-exponential phase were treated with cantharidin (250 µM) in YPD for indicated time. Total RNA was extracted, cDNA was prepared and analyzed by qRT-PCR. *CRG1* transcript levels were normalized to *ACT1*. The simplified diagram of CWI pathway is shown. Slt2 is a kinase, Rlm1 is a transcriptional activator governed by the CWI pathway. (D) A preliminary model describing Crg1-cantharidin interaction. See text for details.

One of the phospholipids manifesting substantial changes in our lipidome analysis was phosphatidylinositol (PI) (Figure S7C). PI is an essential phospholipid with multiple roles in the biosynthesis and metabolism of phosphoinositides (PIP), inositol polyphosphates (IPs), complex sphingolipids and glycerophosphoinositols (GPIs) (Figure 5D) [48]. It has been previously reported that phosphorylated derivatives of PI species (mainly PI(4,5)P) are well-conserved second messengers involved in the regulation of the actin cytoskeleton in Pkc1-dependent manner (Figure 6A) [48], [49]. Therefore, to examine one of the physiological consequences of altered levels of PI, we tested if cantharidin affects the actin cytoskeleton. Microscopy of FITC-phalloidin stained cells revealed that *crg1Δ/Δ* strain treated with 250 µM cantharidin for 1 hour lacked actin patches and displayed highly disorganized actin cables compared to wild type. Overexpression of *CRG1* in *crg1Δ/Δ* strain restored the number of actin patches close to that seen in the wild-type strain without cantharidin (Figure 6B). These results demonstrate that Crg1 is critical for both actin patch and actin cytoskeleton integrity during cantharidin stress. Although, the observed role of Crg1 in cytoskeleton organization might be indirect, in our genome-wide screen (without cantharidin) we found that positive genetic interactions (alleviating) of *CRG1* were significantly enriched for the genes involved in the actin cytoskeleton, bud emergence, and cell polarity (*P*-value <1.0×10^−5^; Figure S8A; Dataset S5). In particular, the deletion of *RVS167*, a well-characterized actin patch and lipid-interacting protein, manifested fitness defects that are suppressed by the deletion of *CRG1* (Figure S8B) [50], [51]. These findings further support the role of Crg1 in actin-related biological process.

### *CRG1* Transcription Is Regulated via the Cell Wall Integrity (CWI) Pathway

Finally, to determine how Crg1 is regulated at the transcriptional level in response to cantharidin, we explored which pathways, if any, are required for cantharidin resistance. Based on our observation that the homozygous deletion strains *slt2Δ/Δ* and *bck1Δ/Δ* (both CWI kinases) are hypersensitive to cantharidin [14], [15], combined with the fact that the promoter region of *CRG1* contains a binding site for Rlm1 (a transcriptional regulator of CWI pathway) [52], we asked if *CRG1* expression is activated by cantharidin via the CWI pathway. We found that deletion of these genes blunted the increase of *CRG1* transcript in response to cantharidin (250 µM) compared to the wild type (Figure 6C), indicating that CWI pathway components are required for *CRG1* expression in the presence of cantharidin. The *CRG1* promoter also contains a binding site for Yap1, a transcription factor required for cadmium tolerance and the oxidative stress response. In contrast to Rlm1 and Slt2, the relative amount of *CRG1* transcript in the *yap1Δ/Δ* mutant was unchanged in the presence of cantharidin (Figure S9A). While these data suggest that Crg1 may be regulated via the CWI pathway and is transcriptionally responsive to numerous cell wall stressing agents (Figure S9B), we did not detect any drastic fitness defects when *crg1* mutants were grown in the presence of cell wall perturbing agents (Figure S9B). However, overexpression of *CRG1* in the *crg1Δ/Δ* mutant did confer resistance to lithium chloride and fenpropimorph, both of which are known perturbants of the cell membrane and other lipid processes (Figure S9C) [53]–[58]. Together these results supports a model in which Crg1 is involved in lipid-related processes that indirectly impinges on cell wall integrity.

## Discussion

In this study we demonstrated that yeast genetic and chemical genome-wide approaches, when combined with rigorous biological follow-up, can effectively characterize a novel gene that, despite being subject to numerous large-scale phenotypic studies, had little functional annotation. Our previous work demonstrated that Crg1, a putative SAM-dependent methyltransferase, was a novel mediator of resistance to the protein phosphatase inhibitor, cantharidin [14]. Here we show that the mechanism of Crg1-cantharidin interaction is through direct methylation of the compound, and that, furthermore, Crg1 plays an essential role in the cellular response to cantharidin-induced lipid alterations.

Our initial observation that cantharidin cytotoxicity is suppressed by overexpression of *CRG1* suggested a specific, although not necessarily direct, cantharidin-Crg1 interaction *in vivo* [14]. Here, we demonstrate that Crg1 is able to interact with cantharidin *in vitro*, resulting in the formation of a methylated cantharidin species. Modification by methylation is known to remove negative charges on diverse molecules which can alter hydrophobicity and modulate cellular pathways and processes. Given the clear phenotype of Crg1-deficient cells and the results from our *in vitro* biochemical characterization of Crg1, we hypothesize that methylation of cantharidin alters its physical properties such that it is no longer harmful to cells. In a manner similar to other methyltransferases that are known to detoxify small molecules [17], [59]–[62], chemical modification of cantharidin provides some insight regarding how its methylation may modify its activity. For example, endothall, an unmethylated, ring-opened form of cantharidin, has been assayed for protein phosphatase inhibition [26] and the methyl, ethyl, and propyl esters of endothall are still potent inhibitors of PP1 and PP2A. Several lactol derivatives of norcantharidin (the anhydride form of endothall) formed by reducing one of the carbonyl groups to a hydroxyl group have been synthesized and characterized. Modification of the free hydroxyl to form methyl, ethyl, and propyl ethers sharply reduced the ability of the drug derivatives to inhibit protein phosphatases. While the unmodified lactol form inhibited PP2A with an IC_50_ of 5 µM, the IC_50_ for the methyl ether lactol form was >1000 µM. Collectively, these observations suggest that methylation of closed-ring forms, not open-ring forms, reduces cellular toxicity. Cantharidin is more sterically hindered than norcantharidin, and as such, we would expect that its equilibrium would favor the closed-ring anhydride form more than that of norcantharidin. Accordingly, we are intrigued by the possibility that the methyl cantharidin product of the reaction catalyzed by Crg1 resembles the closed-ring lactol ether compounds that are less potent inhibitors of both growth and protein phosphatase activity. Further study to elucidate the structure of this product will enhance our understanding of how the methylation of cantharidin by Crg1 facilitates its detoxification.

Looking ahead, it will be interesting to explore if similar mechanisms of cellular detoxification in mammalian cells are mediated by methyltransferases, such as METTL7A or METTL7B, which both share sequence homology to *CRG1*. Interestingly, METTL7B, also known as *ALDI*, was reported to be highly expressed in kidney and liver, and associated with hepatic lipid droplets [63], providing a provocative link between Crg1-like methyltransferases cantharidin toxicity and lipid process.

In addition to characterizing the direct interaction of Crg1 with cantharidin, we investigated cellular pathways of Crg1-mediated cantharidin resistance using cells sensitized with a *crg1* deletion allele. This analysis revealed that genes involved in lipid-related processes are required for survival under cantharidin-induced stress in the absence of *CRG1* (*CHO2*, *OPI3*, *ERG6*, *SAC1*, *ARV1*, *GUP1*, *PER1*, *MOT3*, *DEP1*). Because *CRG1* is both not essential and also shows very few genetic interactions under standard laboratory conditions, the identification of these genes required condition-specific assays. Our chemogenomic data are supported by lipidome-wide analysis, which demonstrated that cantharidin-induced alterations in glycerophospholipids and sphingolipids occur in a *CRG1* gene dose-dependent manner. Specifically, we observed the accumulation of short chain phospholipids in the *crg1Δ/Δ* mutant, suggesting that the drug affects fatty acid elongation in a Crg1-dependent fashion. Consistent with this result, we also observed that overexpression of *CRG1* confers resistance to lipid stressing agents such as lithium ions and the ergosterol inhibitor fenpropimorph (Figure S9C). Resistance to fenpropimorph is acquired by mutations in the fatty acid elongase *FEN1 (ELO2)*, which is known to be involved in sphingolipid biosynthesis [64]. Thus, it will be informative to test if *CRG1* and *FEN1* have overlapping functions in lipid biosynthesis.

Another possible explanation for our observation that cantharidin-induced lipidome alterations can be suppressed by increasing the gene dose of *CRG1* can be found in the transcriptional changes that occur in these strains. Genes involved in methionine biosynthesis are differentially expressed in *CRG1*-overexpressing strains in the presence of the drug compared to wild type and the *crg1Δ/Δ* mutant. This is of particular interest because changes in methionine metabolism can regulate methylation reactions by altering levels of SAM, a methyl donor [65], [66]. For example, Tehlivets *et al.* showed that defects in methionine cycling enzymes result in an imbalance of phospholipid and triacylglycerol synthesis [67], [68]. The mechanisms underlying these relationships are not yet clear, but it is possible that cells sense that the level of SAM is depleted via Crg1 activity, which results in transcriptional changes in methionine biosynthesis genes, in particular, the cystathionine beta-lyase Str3. These findings suggest that overexpression of Crg1 may buffer cantharidin-treated lipidome alterations in part through changes in the methionine cycle.

To define the ‘core’ buffering network to *CRG1* in the presence of cantharidin, we compared the transcriptome and cantharidin SGA profiles. Although we did not find any obvious overlap in GO term biological processes between these datasets, in our cantharidin SGA one of the most sensitive mutants was *MET22* (Figure 4C), a gene with a role in sulfur assimilation and methionine biosynthesis. This gene was also differentially expressed in *CRG1*-overexpressing mutant *vs.* wild-type strain (*P*-value <0.02; Table S2).

Our chemical genomics results were corroborated by traditional SGA analysis. This analysis demonstrated that *CRG1* has an alleviating (suppressing) genetic interaction with *RVS167*. It is well established that a similar phenotype is observed when *RVS167* is deleted in combination with genes involved sphingolipid biosynthesis (e.g. *SUR1*, *SUR2*, *FEN1*, *ELO3* and *IPT1*), implicating sphingolipid biosynthesis in the regulation of the actin cytoskeleton [49], [50], [69]–[71]. Similarly to *S. cerevisiae* and *C. albicans*, studies in the ciliate *Tetrahymena* showed that cantharidin treatment also influences PI metabolism and the actin cytoskeleton [72], demonstrating the conservation of cantharidin-lipid-actin interactions.

Understanding the transcriptional regulation of *CRG1* during cantharidin stress adds many layers to the picture of the complex physiological roles of this methyltransferase. *CRG1* transcription is activated by cantharidin via the conserved MAPK family components of the CWI signaling pathway [52], [73]. Hoon *et al.* previously demonstrated that deletion of *slt2* and *bck1* results in cantharidin sensitivity, suggesting that this pathway is critical for cantharidin resistance [14]. In mammalian cells, several studies have reported that the MAP kinases ERK and JNK are also activated by cantharidin [27], [29], likely as a consequence of the inhibition of protein phosphatases. Moreover, other studies reported that an intact CWI cascade is essential for maintaining lipid homeostasis [74]. It remains to be determined what specific steps are involved in the activation of *CRG1* by cantharidin. One possible scenario is that the CWI pathway is activated by the accumulation of aberrant lipid species in a manner analogous to previous reports that suggest that long chain bases induce the Pkc1-MAPK CWI pathway in yeast [75], [76].

Based on our observations, we propose the following mechanism for Crg1-cantharidin interaction (Figure 6D). Cantharidin treatment inhibits PP2A and PP1, resulting in the perturbation of both lipid homeostasis and actin cytoskeleton organization. This perturbation activates the CWI pathway, which in turn induces of *CRG1* transcription. The resulting Crg1 protein directly methylates cantharidin, alleviating its cytotoxicity and restoring lipid homeostasis, actin cytoskeletal architecture, as well as other cantharidin-associated effects.

In summary, our study demonstrates the value of combining classic biology approaches and chemical genomics with other “omic”-based methods for de-orphaning proteins and elucidating previously unknown mechanisms of therapeutics action.

## Materials and Methods

### Strains, Plasmids, and Growth Conditions

Yeast strains and plasmids used in this study are described in Table S5 and Table S6. Unless otherwise stated, wild-type (wt) strain is BY4743; *crg1Δ/Δ* was derived from BY4743. Yeast cells were grown in YPD (2% yeast extract, 1% peptone, 2% glucose) or in synthetically defined medium, SD (0.67% yeast nitrogen base, 2% glucose, and amino acids). Cantharidin, norcantharidin, cantharidic acid, and fenpropimorph were purchased from Sigma Aldrich (Toronto, Canada). Lithium chloride was purchased from Teknova (Hollister, CA, USA). Cantharidin, cantharidic acid, norcantharidin, and fenpropimorph were dissolved in DMSO and stored at −20°C. The IC_20_ of cantharidin in YPD for wild-type is 250 µM, in SD it is 5 µM, both determined in liquid culture as described [77].

### Site-Directed Mutagenesis of *CRG1*

*CRG1* was amplified from wild-type strain using primers (Table S7) with homology to the vector p426-*GAL1-TAP* at the 5′ end. The amplified *CRG1* and *HindIII* linearized vector were directly co-transformed into a *crg1Δ/Δ* mutant and transformant colonies were selected in synthetic defined media lacking uracil (SD-URA). *CRG1* was cloned downstream of a *GAL1* inducible promoter and in frame with the *TAP* coding sequence. Transformants were screened by PCR and for cantharidin resistance. *CRG1* missense mutants were prepared using the QuickChange Lightning Site-directed mutagenesis kit (Stratagene - Agilent Technologies Company, La Jolla, CA, USA). Clones were sequenced to verify the mutations. To express Crg1, transformants were grown to mid-exponential phase in SD-URA and raffinose (2%), then induced by the addition of galactose to a final concentration of 2%. 30 µM of cantharidin was used to test sensitivity of mutants. Cells were harvested after 3 hours of induction, and Crg1 expression was verified by Western blots of 12% SDS-PAGE gels using *anti*-TAP antibodies (OpenBiosystems – Thermo Fisher Scientific, Huntsville, AL, USA).

### RNA Isolation, cDNA Preparation, and Quantitative Real-Time PCR Analysis

Cells grown to mid-exponential phase in YPD medium were incubated with or without cantharidin (250 µM) for various amounts of time, harvested by centrifugation, frozen in liquid N_2_ and stored at −80°C. RNA was extracted with hot acidic phenol [78] and treated with the Turbo DNA-*free* kit (Ambion – Applied Biosystems, Austin, TX, USA). RNA purity was tested using a spectrophotometer and integrity was evaluated by denaturing gel electrophoresis. First-strand cDNA was synthesized from 1 µg of DNase-treated RNA with 0.5 µg of oligo(dT_12–18_) primers (Invitrogen, Burlington, ON, Canada) using 200 units of Superscript II Reverse transcriptase (Invitrogen, Burlington, ON, Canada). Real-time PCR analysis was conducted with Power SYBR Green PCR master mix (Applied Biosystems, Foster City, CA, USA) and gene-specific primers (Table S7) at a final concentration of 250 nM. qRT-PCR was carried out on a 7900HT Fast system (Applied Biosystems) using Sequence Detection System software version 2.3. Fold change in *CRG1* transcript level normalized to *ACT1* was calculated using the 2^−ΔΔCt^ method. At least three independent replicates of each reaction were performed. Student's t-test was applied for statistical analysis (paired for drug *vs.* DMSO treatments, and unpaired for mutants *vs.* wild type).

### Microarray Analysis

Cells grown to mid-exponential phase in YPD medium were incubated with or without cantharidin (250 µM) for 1 hour, harvested by centrifugation. Isolation of RNA and hybridization to the tiling arrays was performed as described [79], except that actinomycin D was added in a final concentration of 6 µg/mL during cDNA synthesis to prevent antisense artifacts [80]. Two independent replicates were used for the analysis. Hybridization to Affymetrix Tiling Arrays using the GeneChip Fluidics Station 450 (Affymetrix) was followed by the extraction of intensity values for the probes using the GeneChip Operating Software (Affymetrix). Acquisition and quantification of array images were performed using the Affymetrix tiling analysis software (http://www.affymetrix.com/support/developer/downloads/TilingArrayTools/index.affx). The resulting .BAR files containing probe position and intensities were further analyzed by aligning the probes that match the position of the *S. cerevisiae* Genome Database list of defined ORFs (http://downloads.yeastgenome.org/chromosomal_feature/saccharomyces_cerevisiae.gff). The log2 of signal intensity of each ORF was defined as the average across the probes associated with the ORF. Quantile normalized datasets were clustered with a correlation similarity metric and the average linkage method using Cluster 3.0 software (http://bonsai.hgc.jp/~mdehoon/software/cluster/software.htm). The cluster was visualized using TreeView software (http://jtreeview.sourceforge.net/). The significance for differential expression was set as log2 (drug/DMSO) >1 and <−1, *P*-value <0.05 as determined by Student's t test. Significantly up- and downregulated transcripts were further tested for Gene Ontology (GO) biological process enrichment using FunSpec (http://funspec.med.utoronto.ca/) with *P*-value cutoff of 0.01 and multiple testing correction (Bonferroni) (Table S1; Dataset S6). The probability was calculated using a test employing hypergeometric distribution (see below). To detect cantharidin-specific genes the genes involved in ESR [36] were eliminated from the gene-set.

### Expression and Purification of Crg1 Fusion Protein

Purification of 6xHis-Crg1 was performed as previously described [81]. Wild-type yeast strain Y258 carrying a vector pBG1805-*GAL1-CRG1* with a triple affinity tag at C-terminal (His_6_-HA^epitope^-3C^protease site^-ZZ^protein A^) was grown in 660 mL of synthetically defined medium (SD-URA and 2% raffinose) to mid-exponential phase at 30°C. To induce expression of *CRG1* 340 mL of 3x YP (yeast extract and peptone) and 6% galactose was added to a final concentration of 2%. Cells were harvested by centrifugation at 3,000 rpm for 5 min. All steps following harvest were performed at 4°C. Cells were washed with PBS buffer, resuspended in 7 mL of resuspension buffer (20 mM HEPES pH 7.5, 1 M NaCl, 5% glycerol), and lysed using an acid washed Zirconia beads in the presence of protease inhibitors (1 mM Pefablock, 2.5 µg/mL pepstatin A, 2.5 µg/mL leupeptin, 1 mM PMSF). The cell lysate was centrifuged at 20,000 rpm for 45 min, and diluted two fold with binding buffer (20 mM HEPES pH 7.5, 40 mM imidazole, 5% glycerol). 300 µL of Ni Sepharose 6 Fast Flow beads (50% slurry in 20% ethanol) was added to the sample and rotated for 1.5 hours, followed by three washes with 40 mL of wash buffer (20 mM HEPES pH 7.5, 40 mM imidazole, 5% glycerol, 0.5 M NaCl). To elute Crg1 the Ni beads were resuspended in 1 mL of elution buffer (20 mM HEPES pH 7.5, 250 mM imidazole pH 7.7, 5% glycerol, 0.5 M NaCl), and Crg1p was released by rotating the mixture for 15 min at 4°C. The protein was further concentrated with Amicon Ultra tubes (10 K) (Millipore, Etobicoke, ON, Canada) to 100 µL.

Purification of TAP-tagged wild-type and mutant forms of Crg1 was performed in BY4743 carrying p426-*GAL1-CRG1-TAP* as described in Rigaut *et al.* [82]. Cell growth, induction of Crg1 with galactose (2%), and preparation of cell lysates were performed as described for the Crg1-6xHis fusion. 300 µL IgG-agarose was added to the extract and incubated for 2 hours followed by a triple wash with 25 mL of Low Salt and High Salt Wash Buffer (50 mM HEPES pH 7.5, 10% glycerol, 150 mM/750 mM NaCl, 0.1% Tween20). The final wash was performed with 15 mL TEV Cleavage Buffer (10 mM, Tris pH 8.0, 150 mM NaCl, 0.05% Tween20, 10% glycerol, 0.5 mM EDTA, 1 mM DTT). The extract was incubated overnight with 100 U TEV protease (Invitrogen, #12575-015). 1.2 mL CaM Binding Buffer (10 mM Tris pH 8.0, 150 mM NaCl, 0.05% Tween20, 10% glycerol, 1 mM MgOAc, 1 mM imidazole pH 8.0, 2 mM CaCl2, 1 mM DTT) and 2.4 µL 1 M CaCl2 were added to the protein eluates. The eluates were then incubated with 400 µL (50% slurry) Calmodulin Sepharose in 5 mL CaM Binding Buffer for 2 hours, followed by a wash with 25 mL CaM Binding Buffer and elution with 5×200 µL Elution Buffer (10 mM Tris pH 8.0, 150 mM NaCl, 0.05% Tween20, 10% glycerol, 1 mM MgOAc, 1 mM imidazole pH 8.0, 2 mM EGTA, 1 mM DTT). Protein eluates were stored at −20°C in the presence of 50% glycerol. Recovery of Crg1 was determined using Bradford reagent (BioRad Laboratories, Mississauga, ON, Canada) and its integrity and purity was assessed with 12% silver-stained SDS-PAGE gel.

### Preparation and Analysis of Radiolabeled *In Vitro* Crg1 Reactions

*In vitro* enzymatic reactions were prepared with 0.09 µg 6×His-tagged Crg1, 0.2 mM cantharidin dissolved in DMSO (Sigma Aldrich, St. Louis, MO, USA), and 20 µM *S*-adenosyl-[*methyl*-^14^C]methionine (55.8 mCi/mmol) (GE Healthcare, Piscataway, NJ, USA) in 0.1 M sodium phosphate, pH 7.4 with a final volume of 50 µL and 2% DMSO. The complete reactions and relevant controls were incubated at 30°C for 2 hours. Following incubation, the enzymatic reactions were separated by reverse-phase high-performance liquid chromatography (Series II 1090 Liquid Chromatograph, Hewlett Packard, Palo Alto, CA, USA). The chromatography gradient was adapted from [83], except that the flow rate was 1 mL/min. Mobile phase A contained 0.1% trifluoroacetic acid in water and mobile phase B was 0.1% trifluoroacetic acid in acetonitrile. A BetaBasic-18 column (250 mm×4.6 mm; 5-µm particle size) (Thermo, Waltham, MA) was used. 40 µL was injected, 2-minute fractions were collected, and 350 µl of each fraction was mixed with 5 mL of Safety-Solve (Research Products International, Mt. Prospect, IL, USA) before quantification of radioactivity with a LS6500 liquid scintillation counter (Beckman Coulter, La Brea, CA, USA). Each fraction was counted three times for 3 minutes.

Other *in vitro* reactions were prepared in an identical manner with 0.09 µg of either mutant or wild-type TAP-tagged Crg1 and varying concentrations of cantharidin (USB, Cleveland, OH, USA). After incubation at 30°C for 2 hours, 40 µL 2 N HCl was added to each 50 µL reaction. Immediately, 80 µL of this mixture was transferred to a 1.9-cm×9-cm folded piece of filter paper in the neck of a scintillation vial containing 5 mL of Safety-Solve and the vials were capped. After 4 hour incubation at room temperature, the pieces of filter paper were removed from the neck of each vial and the acid-labile volatile radioactivity was quantified with a liquid scintillation counter as described above [84].

### Preparation of *In Vitro* Enzymatic Reactions for Analysis by Mass Spectrometry

*In vitro* enzymatic reactions using unlabeled SAM (Sigma Aldrich) were prepared in a similar manner with 0.09 µg of 6xHis-tagged Crg1, 200 µM cantharidin (Sigma Aldrich), and a SAM concentration of 1.6 mM. These reactions were quenched with addition of 200 µL acetonitrile, and 12.5 µL of 15% ammonium bicarbonate was added to reduce product degradation. After concentration with a vacuum centrifuge and resuspension in 50 µL of water, these reaction mixtures were analyzed by liquid chromatography-tandem mass spectrometry (1100 Series Liquid Chromatograph, Agilent, Santa Clara, CA; QSTAR Elite Mass Spectrometer, Applied Biosystems, Foster City, CA) in positive ionization mode with a Turbo Spray source. 8 µL of sample was injected onto a reverse-phase column (Luna C18 (2), 150 mm×1 mm, 5-µm particle size, Phenomenex, Aschaffenburg, Germany) with a flow rate of 50 µL/min and the following gradient: t = 0–1 min, 2% mobile phase B; t = 10 min, 35% B; t = 14–16 min, 90% B; t = 16.5–35 min, 2% B. Mobile phase A was 0.1% formic acid in 98% water and 2% acetonitrile, while mobile phase B was 0.1% formic acid in 98% acetonitrile and 2% water. Spectra were collected with the instrument information-dependent acquisition mode (full scan: m/z = 70–600, 1.000071 s; 3 product experiments: m/z = 65–500, <2 s per experiment). The following mass spectrometer parameters were used: declustering potential, 85 V; focusing potential, 300 V; declustering potential II, 15 V; ionspray voltage, 5500 V; ion source gas, 45 units; ion source gas II, 5 units; curtain gas, 45 units; collision gas, 7 units.

### Fitness Profiling of Double Deletion Mutants with Cantharidin

A pool of double deletion mutants (*crg1ΔxxxΔ*) was prepared by generating viable double deletion mutants using Synthetic Genetic Array (SGA) technology with *crg1Δ* as a query strain [40] (Text S1). ∼4800 viable double deletion mutant colonies were collected, normalized to 50 OD's/mL and stored at −80°C in media containing 7% DMSO. Two independent pools were generated for the analysis. Each was tested in triplicate. The pooling of the strains was possible due to the presence of strain-specific sequence tags flanking each gene deletion region [5]. The double deletion pool was treated with 30 µM of cantharidin, a dose which inhibits growth of the *crg1* double deletion pool by ∼20%. Fitness analysis using a tag-specific algorithm that takes into account the intensities of each tag in cantharidin-treated cells compared to non-treated cells was performed as described [41]. Hybridization to Affymetrix Gene Chips using GeneChip Fluidics Station 450 (Affymetrix) was followed by the extraction of intensity values for the probes using the GeneChip Operating Software (Affymetrix). The data was quantile normalized, outliers (one standard deviation off) were omitted, and fitness defect scores as the log2 ratio between the mean signal intensities of the control (DMSO) and the drug were calculated for each deletion strain in the pool as previously described [41]. As a control, the relative fitness of the double-gene deletion mutants exhibiting high sensitivity to cantharidin (log2 (drug/DMSO) <−1; *P*-value <0.05) was compared to the relative fitness of the corresponding single gene deletion mutants. For a given double deletion mutants, the resulting interaction was evaluated using comparison of the observed double mutant growth rate to the expected assuming that there is no interaction exists. Log2 ratio <−1 represents those strains with a measurable growth defect (or lethality) and log2 ratio >1 demonstrates resistance to the drug. Cantharidin-specific *CRG1* negative genetic interactors were further verified as individual clones in liquid growth assays and/or by spot dilutions. To evaluate the chemogenomic dataset for statistical significant enrichment for general biological processes (Gene Ontology Slim mapper), we used a standard hypergeometric test, that asses the probability that the intersection of given list with any given functional category occurs by chance. Obtained *P*-values were corrected for multiple testing correction (Bonferroni) by multiplying *P*-values with the number of genes in the test. The probability was calculated as follows: the *P*-value of observing x genes, belonging to the same functional category, is:
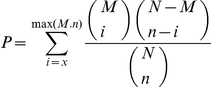
where *M* is the total number of genes involved in a functional category, *n* is the total number of genes in the cluster, and *N* is the total number of yeast ORFs.

### Lipidome Analysis by Liquid Chromatography-Tandem Mass Spectrometry

Cells were grown to mid-exponential phase and treated with cantharidin (250 µM) for 2 hours. Lipids were extracted from 25 OD_600_-equivalent of cells and analyzed as described [43]. All lipid standards were obtained from Avanti Polar Lipids (Alabaster, AL, USA), with the exception of dioctanoyl glycerophosphoethanolamine, which was obtained from Echelon Biosciences (Salt Lake City, UT, USA). Quantification of individual molecular species was carried out using multiple reaction monitoring (MRM) with an Applied Biosystems 4000 Q-Trap mass spectrometer (Applied Biosystems, Foster City, CA, USA). 25 µl of samples were subjected to analysis as described previously [43], [85]. Lipid levels in each sample were normalized to internal standards. For each lipid species, the mean normalized signal from the wild type and mutant strains grown in the presence or absence of cantharidin were calculated. Three independent experiments were used for analysis. Lipid levels were calculated relative to relevant internal standards. The quantities of lipids are expressed as ion intensities relative to the levels without cantharidin, and then converted to a log2 (drug/DMSO) scale. The difference in levels of individual lipid species in DMSO *vs.* cantharidin was determined with Kruskal-Wallis test (Table S4). Similarly, the difference between wild type and mutants was assessed statistically using the Kruskal-Wallis test, with a *P*-value cutoff of 0.05.

### Actin Staining

Cells were grown to mid-exponential phase in YPD media, and treated with and without cantharidin (250 µM) for 1 hour. Cells were then fixed by addition methanol-free formaldehyde (Polysciences, Warrington, PA) to 4% for 1 hour, centrifuged at 3,000 rpm 5 min and washed with PBS buffer three times. Cells were permeabilized with 0.2% Triton X-100 in PBS at 25°C for 15 min, washed with PBS three times and a normalized number of cells were stained with Alexa Fluor 488 phalloidin (Invitrogen, Burlington, ON) in the dark at 25°C for 1 hour. Cells were observed with 100× objective, and fluorescence images were acquired using AxioVision software on an Axiovert 200 M fluorescence microscope (Carl Zeiss) using a 1.5 s exposure for all images. The average number of actin patches per cell was determined by dividing the total number of actin patches per total number of cells (N = 3, n≥300 cells).

## Supporting Information

Dataset S1 Chemical SGA. Fitness profiling of wild-type double deletion mutants (*ura3ΔxxxΔ*) with cantharidin.
(XLS)

Dataset S2 Chemical SGA. Fitness profiling of *crg1* double deletion mutants (*crg1ΔxxxΔ*) with cantharidin.
(XLS)

Dataset S3 Glycerophospholipid and sphingolipid analysis by liquid chromatography-tandem mass spectrometry in *S. cerevisiae*.
(XLS)

Dataset S4 Glycerophospholipid and sphingolipid analysis by liquid chromatography-tandem mass spectrometry in *C. albicans*.
(XLS)

Dataset S5 SGA scores for *crg1* double deletion mutants in the absence of cantharidin.
(XLS)

Dataset S6 Transcriptome profiling of *crg1* mutants upon exposure to cantharidin treatment (250 µM for 1 hour).
(XLS)

Figure S1 (Related to Figure 1.) (A) *CRG1* gene dose is important for cantharidin tolerance. Wt, *crg1Δ/CRG1* heterozygous, *crg1Δ/Δ* homozygous and *CRG1*-overexpressing *crg1Δ/Δ* mutants were assessed in the presence of cantharidin in YPD. Growth curves were obtained by plotting OD_600_ *vs.* time at the tested concentrations of cantharidin. At least three independent replicates were analyzed and the representative growth curves are shown. (B) Cantharidin is more potent in SD media than in YPD. Wt, *crg1Δ/Δ* and *CRG1*-overexpressing *crg1Δ/Δ* mutants were assessed in the presence of cantharidin in SD medium. Dose-response curves were obtained by plotting OD_600_ at saturation point *vs.* tested drug concentrations. Values are means of three independent replicates, and error bars represent standard deviation. (C) Crg1 is important for resistance to the cantharidin analogues cantharidic acid and norcantharidin. Growth of wt, *crg1Δ/Δ* mutant and *crg1Δ/Δ* cells overexpressing *CRG1* was assessed in the presence of cantharidin analogues in YPD. Dose-response curves were obtained by plotting OD_600_ at saturation *vs.* drug concentration. Values are the mean of three independent replicates, and error bars represent the standard deviation.
(PDF)

Figure S2 (Related to Figure 1.) (A) Cantharidin induces *CRG1* transcription in a gene-dose dependent manner. Wt, *crg1Δ/CRG1* heterozygous, *crg1Δ/Δ* homozygous deletion mutants and *CRG1*-overexpressing *crg1Δ/Δ* mutant grown to mid-exponential phase were incubated with or without cantharidin (250 µM) for 1 hour. Total RNA was extracted, cDNA synthesized and the relative abundance of *CRG1* transcript was analyzed by qRT-PCR. Data are the mean of at least three independent experimental replicates, and error bars are the standard deviation. (B) GFP-tagged Crg1 is localized to the cytosol after 1 hour treatment with cantharidin (in low fluorescence medium at 4 µM). Bar, 2.5 µm (see Text S1). (C) Chemical inhibition of protein phosphatases with calyculin A results in the transcriptional induction of *CRG1*. Wt cells grown to mid-exponential phase in YPD were treated with calyculin A (2 µM) for 30, 60 and 120 min. Total RNA was extracted, cDNA synthesized and the relative abundance of *CRG1* transcript was analyzed by qRT-PCR. Data represent the mean of at least three independent experimental replicates, and error bars are the standard deviation. (D) *CRG1* is a stress-responsive methyltransferase. Expression profile of *CRG1* was compared with other yeast genes during 174 diverse environmental stresses [36]. Expression profiles of *CRG1*, *ACT1* and *SSE2* are shown. (E) Crg1 protein level was not altered by mutations in methyltransferase domain. Cells were collected after induction with galactose (2%) for 3 hours. The cell lysates were analyzed by western blotting with anti-TAP antibody. Tubulin was used as an internal loading control and detected by an anti-tubulin antibody.
(PDF)

Figure S3 (Related to Figure 1.) (A) Viability of *crg1* mutants treated with cantharidin for 1 hour. Wt, *crg1Δ/CRG1* heterozygous, *crg1Δ/Δ* homozygous deletion mutants and *CRG1*-overexpressing *crg1Δ/Δ* mutants grown to mid-exponential phase were incubated with or without cantharidin (30 µM and 250 µM, the IC_20_ for *crg1Δ/Δ* and the IC_20_ for wt, respectively) for 1 hour. Cells were normalized to an equivalent OD_600_, 10-fold diluted, spotted onto YPD solid medium and incubated at 30°C. (B) The comparison of GO term Biological processes for the genes that are upregulated and downregulated (log2 ratio >1 and <−1) in a *crg1Δ/Δ* mutant treated with cantharidin (30 µM and 250 µM) for 1 hour. (C) Heat map of the transcriptional profiles of wt, *crg1Δ/Δ* and *CRG1*-overexpressing *crg1Δ/Δ* mutants in response to cantharidin were compared. (D) Hierarchical clustering was used to group all significantly expressed genes (at least two fold) in at least one of the strains in the presence of cantharidin (250 µM). Clusters of genes exhibiting highly similar profiles across the strains are boxed. The overrepresentation of GO Biological processes in these gene clusters are indicated on the right. (E) Cantharidin significantly induces *STR3* transcript levels in *CRG1*-overexpressing *crg1Δ/Δ* mutant. Wt, *crg1Δ/CRG1* heterozygous, *crg1Δ/Δ* homozygous deletion mutants and *CRG1*-overexpressing *crg1Δ/Δ* mutants grown to mid-exponential phase were incubated with or without cantharidin (250 µM) for 1 hour. Total RNA was extracted, cDNA synthesized and the relative abundance of *STR3* transcript was analyzed by qRT-PCR. Data are the mean of at least three independent experimental replicates, and error bars are the standard deviation. (F) Treatment of cells with the non-specific methyltransferase inhibitor SAH increases sensitivity to cantharidin. Wt cells were grown in SC media with or without cantharidin (4 µM) and SAH (50 µM).
(PDF)

Figure S4 (Related to Figure 3.) (A) Silver stained 12% SDS-PAGE of purified TAP-tagged wild-type and mutated Crg1 (see Materials and Methods for details). (B-E) Single ion chromatograms of the major species identified in the spectra shown in Figure 3.
(PDF)

Figure S5 (Related to Figure 4.) (A) Two independent replicates for *crg1Δ/xxxΔ* pools are highly correlated. Only strains with significant log2 (drug/DMSO) (*P*-value <0.05) are included in the analysis. (B) The deletion of *DBF2* suppresses *CRG1* sensitivity to cantharidin. Cells were normalized to an equivalent OD_600_,10-fold diluted, spotted onto synthetic complete medium containing cantharidin (10 µM) and incubated at 30°C. (C) *CRG1*-dependent changes of *DBF2* transcript levels in the presence of cantharidin. Wt, *crg1Δ/CRG1* heterozygous, *crg1Δ/Δ* homozygous deletion mutants and *CRG1*-overexpressing *crg1Δ/Δ* mutants grown to mid-exponential phase were incubated with or without cantharidin (250 µM) for 1 hour. Total RNA was extracted, cDNA synthesized and the relative abundance of *DBF2* transcript was analyzed by qRT-PCR. Data are the mean of at least three independent experimental replicates, and error bars are the standard deviation.
(PDF)

Figure S6 (Related to Figure 5.) (A) GO term enrichment analysis of cantharidin-specific *CRG1*-interactors before and after MDR gene filtering. Only the terms with significant *P*-values (<0.05) are shown. Bonferroni correction was applied to the MDR-filtered terms. (B) Representative images of cells stained with Nile Red for lipid droplets. Cells were grown at 30°C for 42 hours to reach stationary phase, and inoculated into fresh medium with cantharidin (250 µM) for 2 hours. Cells were fixed and stained with Nile Red. Bar, 2.5 µm. (C) Sterol species are not affected by the deletion of *CRG1* and cantharidin treatment (see Materials and Methods for details).
(PDF)

Figure S7 (Related to Figure 5.) The *C. albicans* functional homolog of *CRG1*, *orf19.633*, has a role in lipid homeostasis. (A) *orf19.633*, a putative methyltransferase, is required for cantharidin resistance. Fitness of wt (SN87) and *orf19.633Δ/Δ* mutant were measured in liquid YPD medium in the presence of cantharidin (2 mM, the IC_20_ for wt). Dose-response growth curves were obtained by plotting OD_600_ at saturation point in liquid versus tested drug concentrations. (B) Comparative lipidomics of cantharidin-treated *C. albicans* wild type and *orf19.633Δ/Δ* mutant. The cells were treated with 2 mM cantharidin (2 hours) and further prepared as described in Figure 5. Only those lipid species with significant changes in their abundance between wt and *orf19.633Δ/Δ* mutant are shown (*P*-value <0.05, Student's t-test). (C) PI species are significantly affected by cantharidin in Crg1-dependent manner. Cells were prepared as described in Figure 5. Only those PI species with significant changes in their abundance between wt and *crg1Δ/Δ* mutant are shown (*P*-value <0.05, Student's t-test). The PI content of *CRG1*-overexpressing mutant is not significantly different form the one in wild-type strain (*P*-value >0.05).
(PDF)

Figure S8 (Related to Figure 5.) The genetic interactome of *CRG1* reveals functional networks required for buffering the absence of *CRG1* in standard laboratory condition. (A) Genetic interactors of *CRG1* identified through SGA analysis. The double deletion mutants *crg1ΔxxxΔ* were generated by SGA as previously described [86]. The double and single mutant fitness (based on colony sizes) from two independent SGA screens were used to quantify the strength of genetic interaction between *CRG1* and other gene. Quantification was performed using the quantitative SGA scoring algorithm as described in [87]. The significant genetic interactors (*P*-value <0.05) common between two SGA screens were considered as hits. The mutants with SGA score >0.08 are significant positive or alleviating interactors, the ones with <0.08 are significant negative or aggravating interactors. (B) Deletion of *CRG1* suppresses fitness defect of *rvs167Δ* mutant. Double and single mutants were grown in synthetic complete (SC) medium to the saturation. *ura3Δ his3Δ* is used as the control strain.
(PDF)

Figure S9 (Related to Figure 6.) (A) Transcriptional activator Yap1 is not required to activate *CRG1* transcription during cantharidin stress. Sample treatment and qRT-PCR analysis were performed as described in Figure 1B. (B) *CRG1* is transcriptionally activated by cell wall perturbing agents, however, it is not required for the growth in their presence. qRT-PCR analysis was performed as described in Figure 1B. Cells normalized to equal OD_600_ were 10-fold diluted, spotted onto solid YPD medium containing various cell wall and membrane perturbing agents, and incubated at 30°C for 2–3 days. (C) Overexpression of *CRG1* confers resistance to fenpropimorph. Mid-exponentially grown cells were normalized to equal OD_600_ were 10-fold diluted, spotted onto solid YPD medium containing fenpropimorph (250 µM), and incubated at 30°C for 2–3 days.
(PDF)

Table S1 Significantly enriched (*P*-value <1.0×10^−6^) Gene Ontology Biological processes for significantly up- and downregulated genes (log2 >1 and <−1) in the presence of cantharidin.
(XLSX)

Table S2 Differentially expressed genes of methionine biosynthesis in *crg1* mutants under cantharidin stress.
(XLSX)

Table S3 Cantharidin-specific genetic interactors of *CRG1*.
(XLSX)

Table S4 Statistical analysis (Kruskal-Wallis test) of lipid abundance in *crg1* mutants exposed to cantharidin.
(XLSX)

Table S5 Yeast strains used in this study.
(DOC)

Table S6 Plasmids used in this study.
(DOC)

Table S7 Primers used in this study.
(DOC)

Text S1 Supporting Materials and Methods.
(DOC)
