# Supplementary figures and images for: A Systems Biology Approach Reveals the Role of a Novel Methyltransferase in Response to Chemical Stress and Lipid Homeostasis
Source: PLoS Genet. 2011 Oct 20;7(10):e1002332. doi: 10.1371/journal.pgen.1002332 (PMC3197675; doi:10.1371/journal.pgen.1002332)

Figure S1

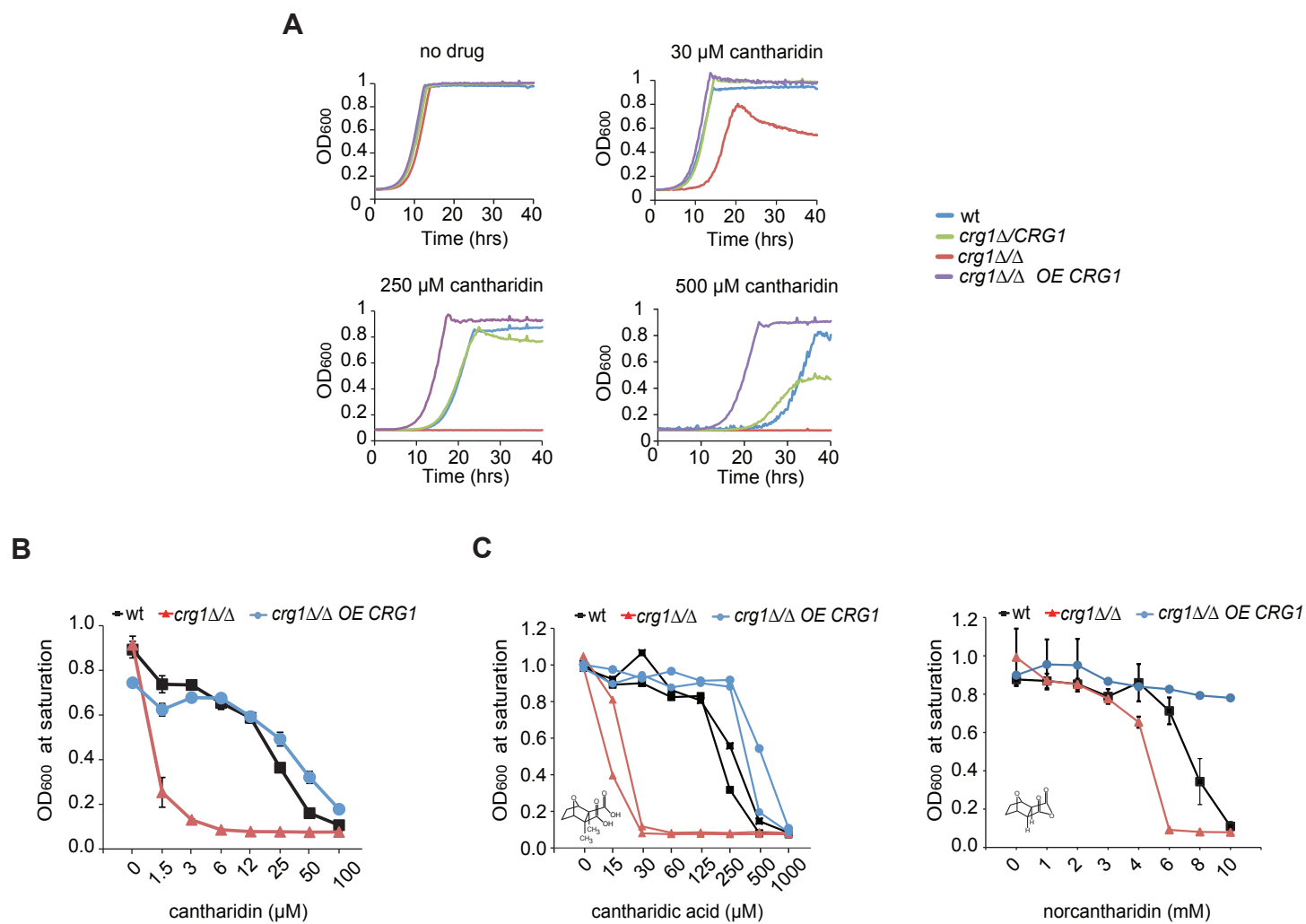

Supplement: Figure S1 — (Related to Figure 1.) (A) CRG1 gene dose is important for cantharidin tolerance. Wt, crg1Δ/CRG1 heterozygous, crg1Δ/Δ homozygous and CRG1-overexpressing crg1Δ/Δ mutants were assessed in the presence of cantharidin in YPD. Growth curves were obtained by plotting OD600 vs. time at the tested concentrations of cantharidin. At least three independent replicates were analyzed and the representative growth curves are shown. (B) Cantharidin is more potent in SD media than in YPD. Wt, crg1Δ/Δ and CRG1-overexpressing crg1Δ/Δ mutants were assessed in the presence of cantharidin in SD medium. Dose-response curves were obtained by plotting OD600 at saturation point vs. tested drug concentrations. Values are means of three independent replicates, and error bars represent standard deviation. (C) Crg1 is important for resistance to the cantharidin analogues cantharidic acid and norcantharidin. Growth of wt, crg1Δ/Δ mutant and crg1Δ/Δ cells overexpressing CRG1 was assessed in the presence of cantharidin analogues in YPD. Dose-response curves were obtained by plotting OD600 at saturation vs. drug concentration. Values are the mean of three independent replicates, and error bars represent the standard deviation. (PDF) [file pgen.1002332.s007.pdf]

Figure S2

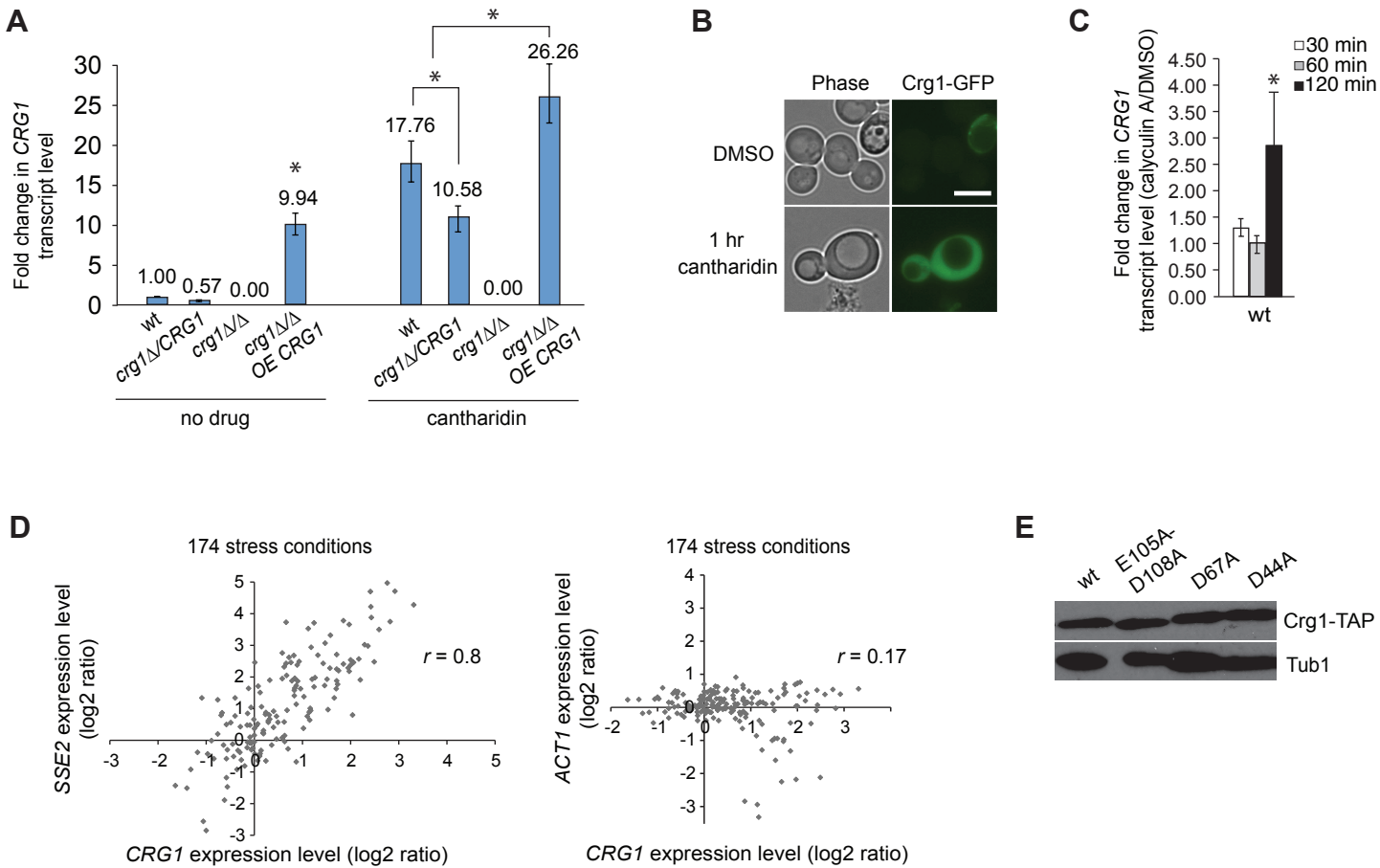

Supplement: Figure S2 — (Related to Figure 1.) (A) Cantharidin induces CRG1 transcription in a gene-dose dependent manner. Wt, crg1Δ/CRG1 heterozygous, crg1Δ/Δ homozygous deletion mutants and CRG1-overexpressing crg1Δ/Δ mutant grown to mid-exponential phase were incubated with or without cantharidin (250 µM) for 1 hour. Total RNA was extracted, cDNA synthesized and the relative abundance of CRG1 transcript was analyzed by qRT-PCR. Data are the mean of at least three independent experimental replicates, and error bars are the standard deviation. (B) GFP-tagged Crg1 is localized to the cytosol after 1 hour treatment with cantharidin (in low fluorescence medium at 4 µM). Bar, 2.5 µm (see Text S1). (C) Chemical inhibition of protein phosphatases with calyculin A results in the transcriptional induction of CRG1. Wt cells grown to mid-exponential phase in YPD were treated with calyculin A (2 µM) for 30, 60 and 120 min. Total RNA was extracted, cDNA synthesized and the relative abundance of CRG1 transcript was analyzed by qRT-PCR. Data represent the mean of at least three independent experimental replicates, and error bars are the standard deviation. (D) CRG1 is a stress-responsive methyltransferase. Expression profile of CRG1 was compared with other yeast genes during 174 diverse environmental stresses [36]. Expression profiles of CRG1, ACT1 and SSE2 are shown. (E) Crg1 protein level was not altered by mutations in methyltransferase domain. Cells were collected after induction with galactose (2%) for 3 hours. The cell lysates were analyzed by western blotting with anti-TAP antibody. Tubulin was used as an internal loading control and detected by an anti-tubulin antibody. (PDF) [file pgen.1002332.s008.pdf]

Figure S3

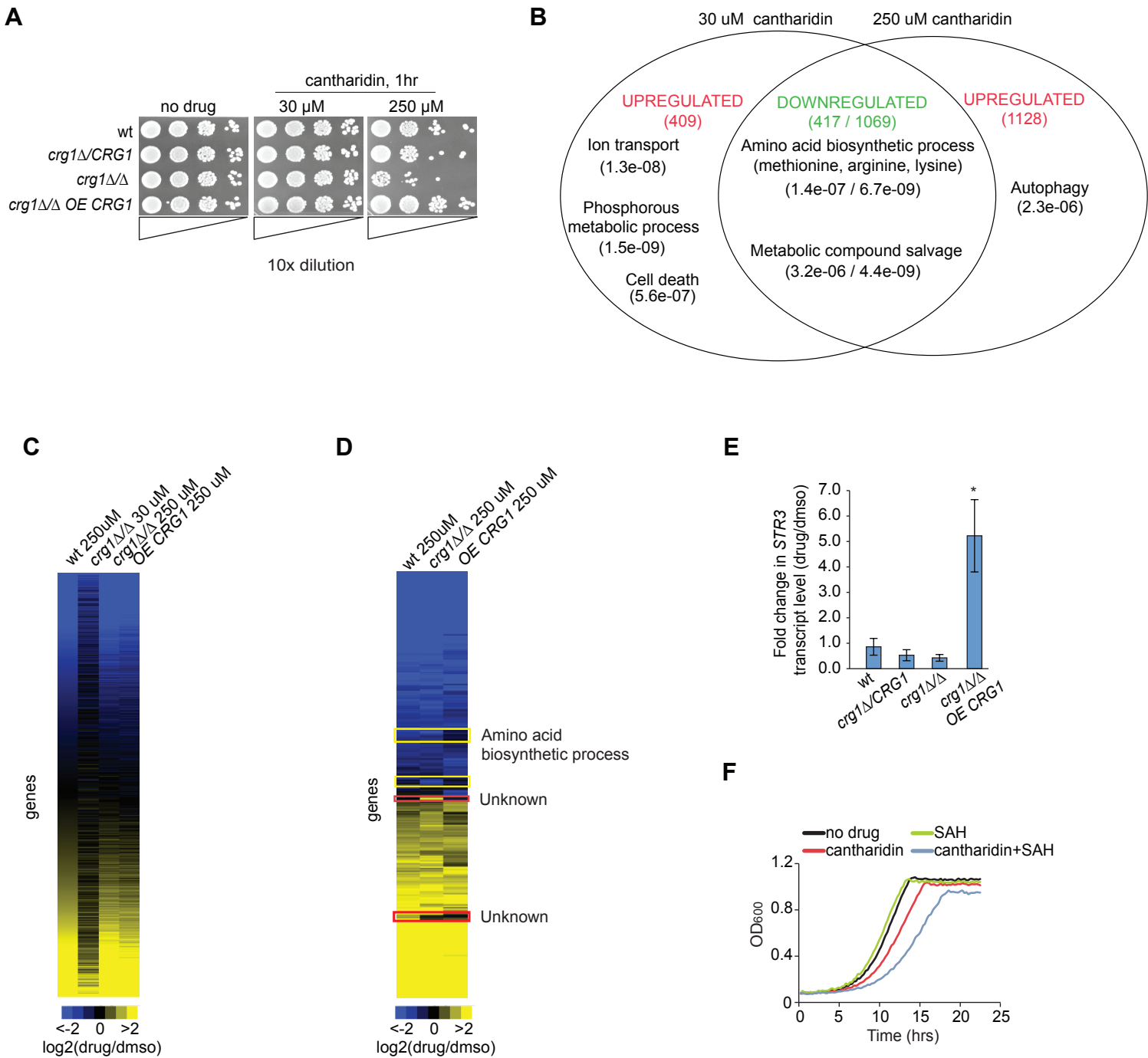

Supplement: Figure S3 — (Related to Figure 1.) (A) Viability of crg1 mutants treated with cantharidin for 1 hour. Wt, crg1Δ/CRG1 heterozygous, crg1Δ/Δ homozygous deletion mutants and CRG1-overexpressing crg1Δ/Δ mutants grown to mid-exponential phase were incubated with or without cantharidin (30 µM and 250 µM, the IC20 for crg1Δ/Δ and the IC20 for wt, respectively) for 1 hour. Cells were normalized to an equivalent OD600, 10-fold diluted, spotted onto YPD solid medium and incubated at 30°C. (B) The comparison of GO term Biological processes for the genes that are upregulated and downregulated (log2 ratio >1 and <−1) in a crg1Δ/Δ mutant treated with cantharidin (30 µM and 250 µM) for 1 hour. (C) Heat map of the transcriptional profiles of wt, crg1Δ/Δ and CRG1-overexpressing crg1Δ/Δ mutants in response to cantharidin were compared. (D) Hierarchical clustering was used to group all significantly expressed genes (at least two fold) in at least one of the strains in the presence of cantharidin (250 µM). Clusters of genes exhibiting highly similar profiles across the strains are boxed. The overrepresentation of GO Biological processes in these gene clusters are indicated on the right. (E) Cantharidin significantly induces STR3 transcript levels in CRG1-overexpressing crg1Δ/Δ mutant. Wt, crg1Δ/CRG1 heterozygous, crg1Δ/Δ homozygous deletion mutants and CRG1-overexpressing crg1Δ/Δ mutants grown to mid-exponential phase were incubated with or without cantharidin (250 µM) for 1 hour. Total RNA was extracted, cDNA synthesized and the relative abundance of STR3 transcript was analyzed by qRT-PCR. Data are the mean of at least three independent experimental replicates, and error bars are the standard deviation. (F) Treatment of cells with the non-specific methyltransferase inhibitor SAH increases sensitivity to cantharidin. Wt cells were grown in SC media with or without cantharidin (4 µM) and SAH (50 µM). (PDF) [file pgen.1002332.s009.pdf]

Figure S4

A

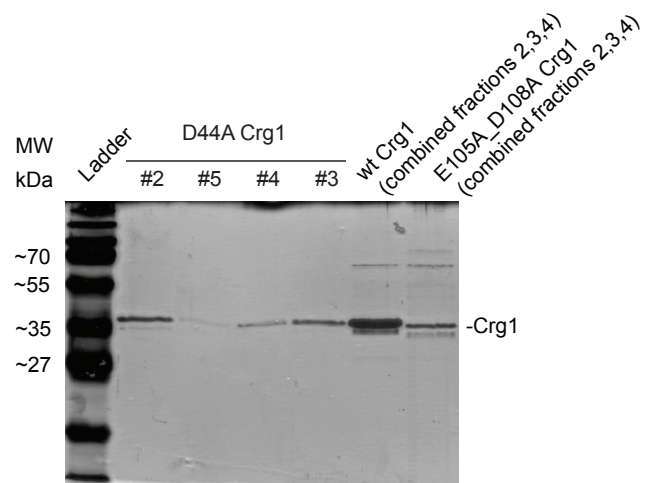

B

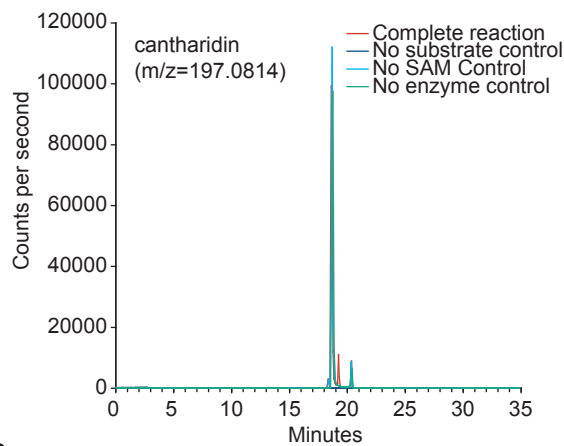

C

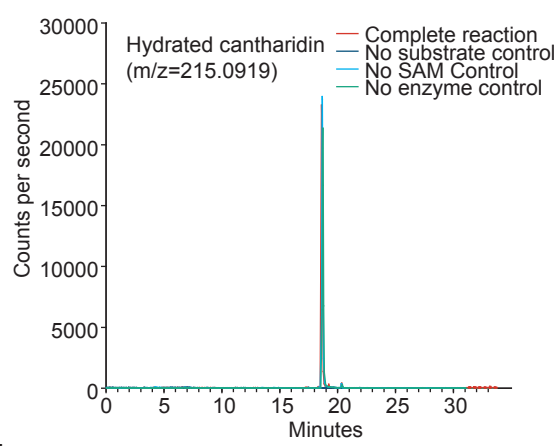

D

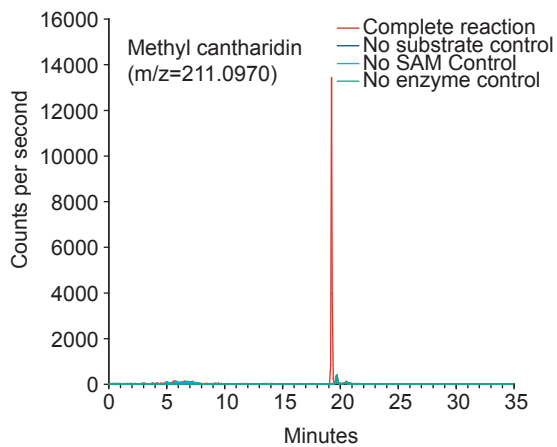

E

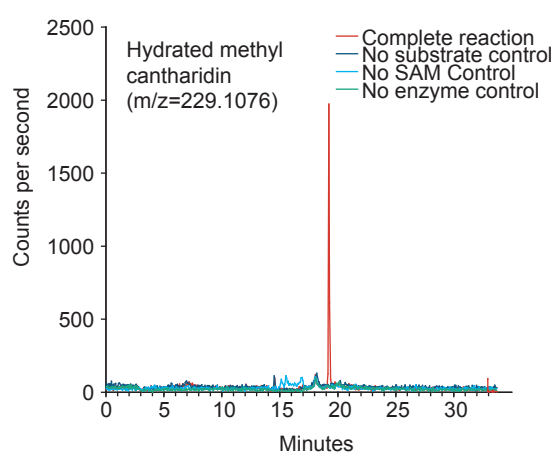

Supplement: Figure S4 — (Related to Figure 3.) (A) Silver stained 12% SDS-PAGE of purified TAP-tagged wild-type and mutated Crg1 (see Materials and Methods for details). (B-E) Single ion chromatograms of the major species identified in the spectra shown in Figure 3. (PDF) [file pgen.1002332.s010.pdf]

Figure S5

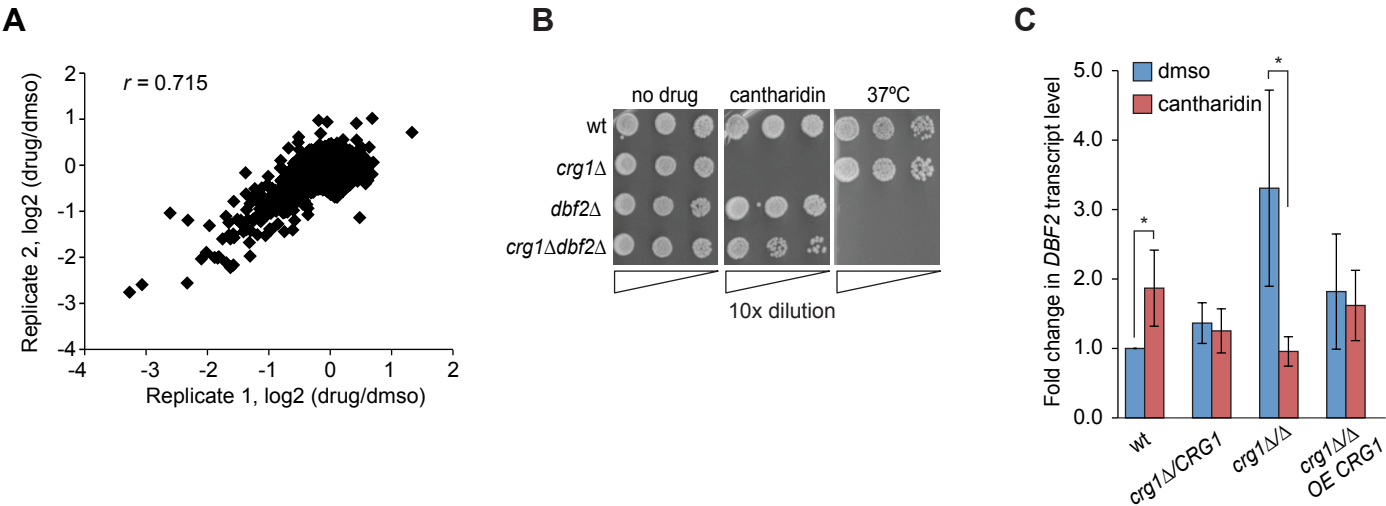

Supplement: Figure S5 — (Related to Figure 4.) (A) Two independent replicates for crg1Δ/xxxΔ pools are highly correlated. Only strains with significant log2 (drug/DMSO) (P-value <0.05) are included in the analysis. (B) The deletion of DBF2 suppresses CRG1 sensitivity to cantharidin. Cells were normalized to an equivalent OD600,10-fold diluted, spotted onto synthetic complete medium containing cantharidin (10 µM) and incubated at 30°C. (C) CRG1-dependent changes of DBF2 transcript levels in the presence of cantharidin. Wt, crg1Δ/CRG1 heterozygous, crg1Δ/Δ homozygous deletion mutants and CRG1-overexpressing crg1Δ/Δ mutants grown to mid-exponential phase were incubated with or without cantharidin (250 µM) for 1 hour. Total RNA was extracted, cDNA synthesized and the relative abundance of DBF2 transcript was analyzed by qRT-PCR. Data are the mean of at least three independent experimental replicates, and error bars are the standard deviation. (PDF) [file pgen.1002332.s011.pdf]

Figure S6

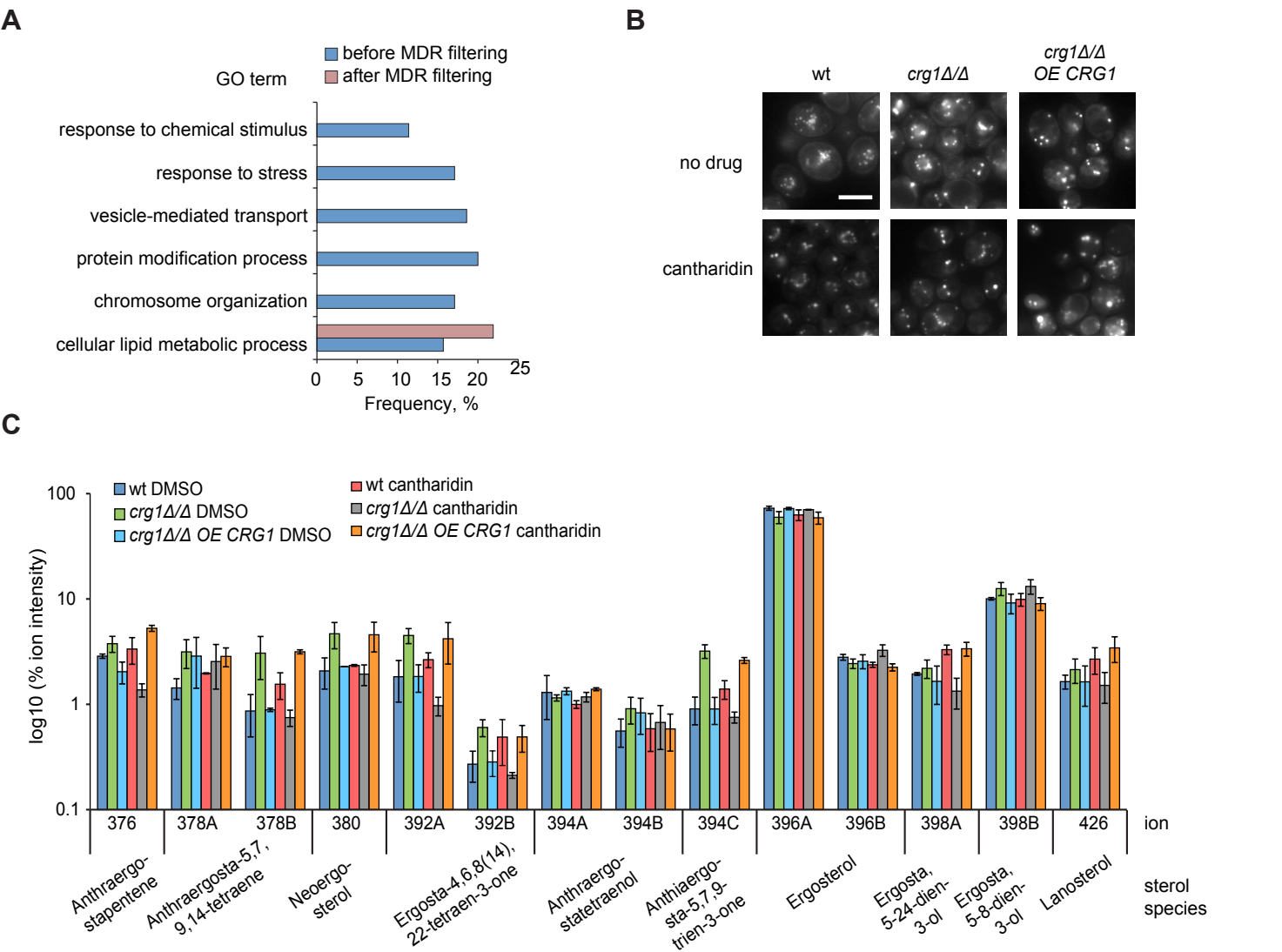

Supplement: Figure S6 — (Related to Figure 5.) (A) GO term enrichment analysis of cantharidin-specific CRG1-interactors before and after MDR gene filtering. Only the terms with significant P-values (<0.05) are shown. Bonferroni correction was applied to the MDR-filtered terms. (B) Representative images of cells stained with Nile Red for lipid droplets. Cells were grown at 30°C for 42 hours to reach stationary phase, and inoculated into fresh medium with cantharidin (250 µM) for 2 hours. Cells were fixed and stained with Nile Red. Bar, 2.5 µm. (C) Sterol species are not affected by the deletion of CRG1 and cantharidin treatment (see Materials and Methods for details). (PDF) [file pgen.1002332.s012.pdf]

Figure S7

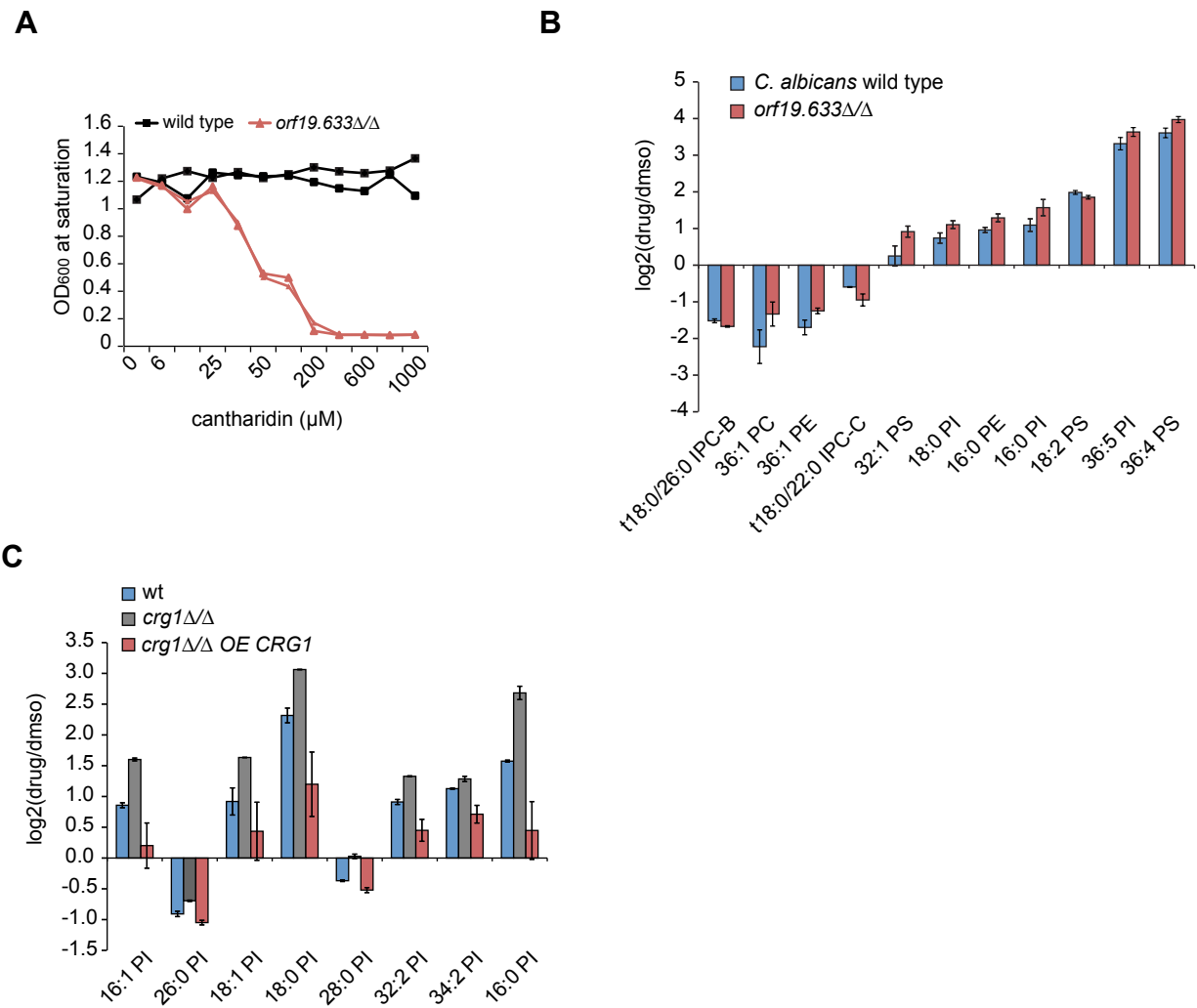

Supplement: Figure S7 — (Related to Figure 5.) The C. albicans functional homolog of CRG1, orf19.633, has a role in lipid homeostasis. (A) orf19.633, a putative methyltransferase, is required for cantharidin resistance. Fitness of wt (SN87) and orf19.633Δ/Δ mutant were measured in liquid YPD medium in the presence of cantharidin (2 mM, the IC20 for wt). Dose-response growth curves were obtained by plotting OD600 at saturation point in liquid versus tested drug concentrations. (B) Comparative lipidomics of cantharidin-treated C. albicans wild type and orf19.633Δ/Δ mutant. The cells were treated with 2 mM cantharidin (2 hours) and further prepared as described in Figure 5. Only those lipid species with significant changes in their abundance between wt and orf19.633Δ/Δ mutant are shown (P-value <0.05, Student's t-test). (C) PI species are significantly affected by cantharidin in Crg1-dependent manner. Cells were prepared as described in Figure 5. Only those PI species with significant changes in their abundance between wt and crg1Δ/Δ mutant are shown (P-value <0.05, Student's t-test). The PI content of CRG1-overexpressing mutant is not significantly different form the one in wild-type strain (P-value >0.05). (PDF) [file pgen.1002332.s013.pdf]

Figure S8

A

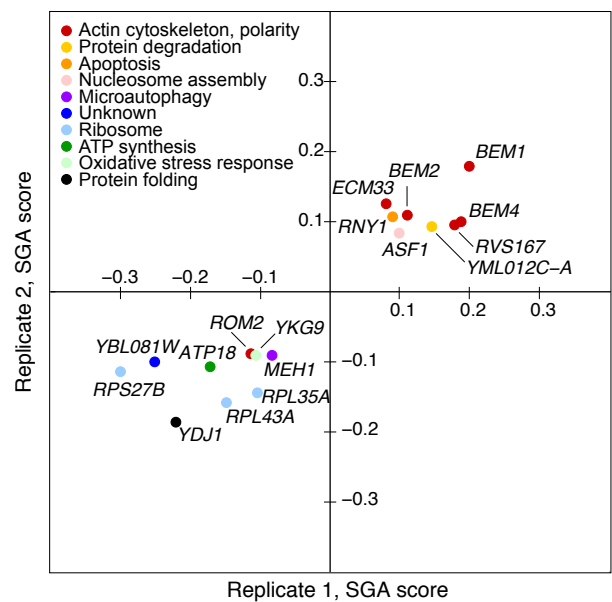

B

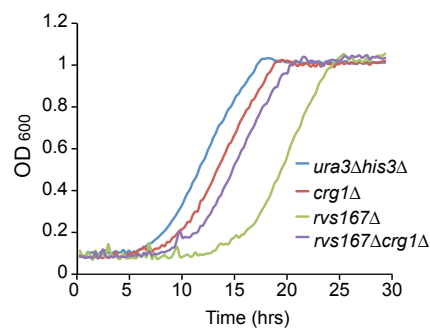

Supplement: Figure S8 — (Related to Figure 5.) The genetic interactome of CRG1 reveals functional networks required for buffering the absence of CRG1 in standard laboratory condition. (A) Genetic interactors of CRG1 identified through SGA analysis. The double deletion mutants crg1ΔxxxΔ were generated by SGA as previously described [86]. The double and single mutant fitness (based on colony sizes) from two independent SGA screens were used to quantify the strength of genetic interaction between CRG1 and other gene. Quantification was performed using the quantitative SGA scoring algorithm as described in [87]. The significant genetic interactors (P-value <0.05) common between two SGA screens were considered as hits. The mutants with SGA score >0.08 are significant positive or alleviating interactors, the ones with <0.08 are significant negative or aggravating interactors. (B) Deletion of CRG1 suppresses fitness defect of rvs167Δ mutant. Double and single mutants were grown in synthetic complete (SC) medium to the saturation. ura3Δ his3Δ is used as the control strain. (PDF) [file pgen.1002332.s014.pdf]

Figure S9

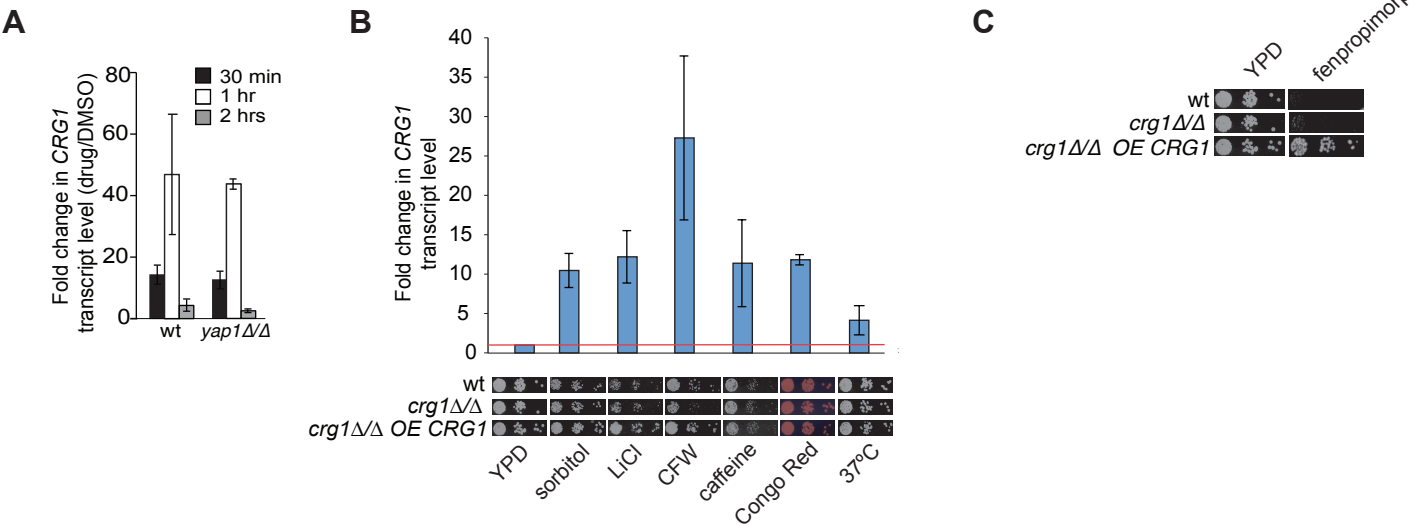

Supplement: Figure S9 — (Related to Figure 6.) (A) Transcriptional activator Yap1 is not required to activate CRG1 transcription during cantharidin stress. Sample treatment and qRT-PCR analysis were performed as described in Figure 1B. (B) CRG1 is transcriptionally activated by cell wall perturbing agents, however, it is not required for the growth in their presence. qRT-PCR analysis was performed as described in Figure 1B. Cells normalized to equal OD600 were 10-fold diluted, spotted onto solid YPD medium containing various cell wall and membrane perturbing agents, and incubated at 30°C for 2–3 days. (C) Overexpression of CRG1 confers resistance to fenpropimorph. Mid-exponentially grown cells were normalized to equal OD600 were 10-fold diluted, spotted onto solid YPD medium containing fenpropimorph (250 µM), and incubated at 30°C for 2–3 days. (PDF) [file pgen.1002332.s015.pdf]
