# Supplementary material for: A Systems Biology Approach Reveals the Role of a Novel Methyltransferase in Response to Chemical Stress and Lipid Homeostasis
Source: PLoS Genet. 2011 Oct 20;7(10):e1002332. doi: 10.1371/journal.pgen.1002332 (PMC3197675; doi:10.1371/journal.pgen.1002332)
Supplement: Table S5 — Yeast strains used in this study. (DOC) [file pgen.1002332.s020.doc]

**Table S5. Yeast strains used in this study.**

| **Strain** | **Genotype and Source** |
| --- | --- |
| BY4743 (wild-type) | *MAT*a/α *his3*Δ*1/his3*Δ*1 leu2*Δ*0/leu2*Δ*0 LYS2/lys2*Δ*0 met15*Δ*0/MET15 ura3*Δ*0/ura3*Δ*0* [1] |
| *crg1Δ* | Isogenic to BY4743, except for *crg1::KanMX crg1::KanMX* [2] |
| *bck1Δ* | Isogenic to BY4743, except for *bck1::KanMX bck1::KanMX* [2] |
| *slt2Δ* | Isogenic to BY4743, except for *slt2::KanMX slt2::KanMX* [2] |
| *rlm1Δ* | Isogenic to BY4743, except for *rlm1::KanMX rlm1::KanMX* [2] |
| *yap1Δ* | Isogenic to BY4743, except for *yap1::KanMX yap1::KanMX* [2] |
| *glc7Δ het* | Isogenic to BY4743, except for *GLC7/glc7::KanMX* [2] |
| BY4741 | *MATa his3Δ1 leu2Δ0 met15Δ0 ura3Δ0;* EUROSCARF collection |
| *CRG1-GFP* | Isogenic to BY4741, except *CRG1-GFP::NatMX;* this study |
| SGA strain | *Mata can1Δ::MFA1pr-HIS3 lyp1Δ ura3Δ0 leu2Δ0 his3Δ1 met15Δ0* [3] |
| Y258 | *Mat*a, *pep4-3, his4-580, ura3-52, leu2-3, 112;* Open Biosystems |
| CaLC238 | SN87, derivative of SC5314 *leu2Δ/leu2Δ his1Δ/his1Δ URA3/ura3::imm434 IRO1/iro1::imm434* [4] |
| CaLC941 | Isogenic to SN87, except *orf19.633::CdHIS1/orf19.633::CmLEU2;* Leah Cowen |
|  | **Collection of deletion mutants** |
| *ArrayORFΔ* | *MATa orfΔ::KanMX4 LYS2 his3Δ1 leu2Δ0 met15Δ0 ura3Δ0* [2] |

**References**

1. Brachmann CB, Davies A, Cost GJ, Caputo E, [Li J](http://www.ncbi.nlm.nih.gov/pubmed?term="Li J"%5BAuthor%5D), *et al.* (1998) Designer deletion strains derived from *Saccharomyces cerevisiae* S288C: a useful set of strains and plasmids for PCR-mediated gene disruption and other applications. Yeast 14: 115-132.

2. Giaever G, Chu AM, Ni L, Connelly C, Riles L, *et al.* (2002) Functional profiling of the *Saccharomyces cerevisiae* genome. Nature 418: 387-391.

3. Costanzo M, Baryshnikova A, Bellay J, Kim Y, Spear ED, *et al.* (2010) The genetic landscape of a cell. Science 327: 425-431.

4. Noble SM, [Johnson AD](http://www.ncbi.nlm.nih.gov/pubmed?term="Johnson AD"%5BAuthor%5D) (2005) Strains and strategies for large-scale gene deletion studies of the diploid human fungal pathogen Candida albicans. Eukaryot Cell 4: 298-309.
