# Supplementary material for: A Systems Biology Approach Reveals the Role of a Novel Methyltransferase in Response to Chemical Stress and Lipid Homeostasis
Source: PLoS Genet. 2011 Oct 20;7(10):e1002332. doi: 10.1371/journal.pgen.1002332 (PMC3197675; doi:10.1371/journal.pgen.1002332)
Supplement: Table S6 — Plasmids used in this study. (DOC) [file pgen.1002332.s021.doc]

**Plasmids used in this study**

Standard methodologies were used to generate the plasmids containing c-terminal- tagged *CRG1-6XHis*. DNA sequences were amplified from genomic DNA and cloned under the control of *GAL1* promoter into the plasmid BG1805.

**Table S6. Plasmids used in this study.**

| **Plasmid** | **Description and Source** |
| --- | --- |
| YEp351 | *2 μm, LEU2,* Charles Boone |
| YEp351-*CRG1* | *CRG1* in YEp352, Charles Boone |
| BG1805 | *2 μm, URA3, GAL1prom,* triple affinity tag *(His6-HAepitope-3Cprotease site-ZZproteinA)* at C-terminal, this study |
| BG1805*-CRG1* | *CRG1* in BG1805, Open Biosystems |
| P426-*GAL1* | *2 μm, URA3, GAL1prom, TAP* tag at C-terminal, Grant Brown |
| P426-*GAL1-CRG1* | *2 μm, URA3, GAL1prom, TAP* tag fused to *CRG1* at C-terminal, this study |
| E105A_D108A | *2 μm, URA3, GAL1prom, TAP* tag fused to *crg1-E105A,D108A* at C-terminal, this study |
| D67A | *2 μm, URA3, GAL1prom, TAP* tag fused to *crg1-D67A* at C-terminal, this study |
| D44A | *2 μm, URA3, GAL1prom, TAP* tag fused to *crg1-D44A* at C-terminal, this study |
| C119Y | *2 μm, URA3, GAL1prom, TAP* tag fused to *crg1-C119Y* at C-terminal, this study |
| G96A | *2 μm, URA3, GAL1prom, TAP* tag fused to *crg1-G96A* at C-terminal, this study |
