# Supplementary material for: A Systems Biology Approach Reveals the Role of a Novel Methyltransferase in Response to Chemical Stress and Lipid Homeostasis
Source: PLoS Genet. 2011 Oct 20;7(10):e1002332. doi: 10.1371/journal.pgen.1002332 (PMC3197675; doi:10.1371/journal.pgen.1002332)
Supplement: Table S7 — Primers used in this study. (DOC) [file pgen.1002332.s022.doc]

**Table S7. Primers used in this study**.

| **Primer name** | **Purpose** | **Oligonucleotide sequence (5’-3’)** |
| --- | --- | --- |
| frwCRG1 | p426-*GAL1-CRG1-TAP* | CTGCAGGAATTCGATATCATGCCTAAAACTAGTTATTTA |
| revCRG1 | p426-*GAL1-CRG1-TAP* | CTTTTCCATATCGATAAGTTCTCTTTTCCTACATAAGTA |
| frwE105A_D108A | *CRG1* mutagenesis | GCATTCGACCAGCAAGTGTAGCTATGGTTATTTCAGC |
| revE105A_D108A | *CRG1* mutagenesis | GCTGAAATAACCATATCTACACTTGCTGGTCGAATGC |
| frwD67A | *CRG1* mutagenesis | GGAAGTGATTGGGATTGCTCCTTCTTCTGCTATG |
| revD67A | *CRG1* mutagenesis | CATAGCAGAAGAAGGAGCAATCCCAATCACTTCC’ |
| frwD44A | *CRG1* mutagenesis | CGCAAAAGTTTGGTTGCTATTGGATGTGGCACA |
| revD44A | *CRG1* mutagenesis | TGTGCCACATCCAATAGCAACCAAACTTTTGCG |
| frwG96A | *CRG1* mutagenesis | ATTAATGCGCCTGCTGAAGATTTATCC |
| revG96A | *CRG1* mutagenesis | GGATAAATCTTCAGCAGGCGCATTAAT’ |
| frwACT1_qPCR | qRT-PCR | TGTGATGTCGATGTCCGTAAG |
| revACT1_qPCR | qRT-PCR | CGGTGATTTCCTTTTGCATT |
| frwCRG1_qPCR | qRT-PCR | GAAAGGCTGTTTCAGCAGGT’ |
| revCRG1_qPCR | qRT-PCR | ATTCAAGGCTTCGGGAAAGT |
| frwCRG1-GFP | Crg1-GFP tag | TTGAATGTACCTTTAAAAATAGAGTGGTCAACGTTTTATTACTTATGTAGGAAAAGAGAAGGTGAAGCTCAAAAACTTAAT |
| revCRG1-GFP | Crg1-GFP tag | TTGACGGTTTTAAATTACTCCCAACTGCGCAACAACACCTTATTTTCTTTTTGCCATATTGCTGACGGTATCGATAAGCCT |
