# Supplementary material for: A Systems Biology Approach Reveals the Role of a Novel Methyltransferase in Response to Chemical Stress and Lipid Homeostasis
Source: PLoS Genet. 2011 Oct 20;7(10):e1002332. doi: 10.1371/journal.pgen.1002332 (PMC3197675; doi:10.1371/journal.pgen.1002332)
Supplement: Text S1 — Supporting Materials and Methods. (DOC) [file pgen.1002332.s023.doc]

**Text S1. Supporting Materials and Methods**

**Construction of a Crg1-GFP Fusion Protein**

To construct a chromosomally GFP-tagged Crg1 protein, a pair of oligonucleotides (Table S7) with homology to the desired chromosomal insertion site at the 5’end of each primer and homology to a vector containing the GFP tag at the 3’end was used to amplify the GFP tag and *NATMX* resistance marker from a plasmid template [88], and the resulting PCR products were transformed directly into wild-type BY4741 using the high-efficiency lithium acetate transformation protocol [89]. Transformants were selected on medium containing nourseothricin and assessed by genomic DNA PCR with primers specific for *GFP* and *CRG1*. For fluorescence microscopy, cells were used without fixation. Cells were grown to mid-exponential phase in low-fluorescence synthetically complete medium (MP Biomedicals, LLC, Solon, Ohio, USA) and incubated with or without cantharidin (4 µM). Cells were visualized with a 100x objective on an Axiovert 200M fluorescence microscope (Carl Zeiss). Images were acquired with a Zeiss HRM digital camera using AxioVision software.

**Synthetic Genetic Array (SGA) and Quantification of SGA score**

Two independent SGA screens of *crg1Δ* as the query strain against the non-essential gene deletion array (4293 strains) were performed as previously described [86]. Computer-based quantification analysis of digital images was used to identify double mutant strains (*crg1ΔxxxΔ*) exhibiting growth differences relative to a control set of double deletion mutants (*ura3∆xxx∆*) [87]. The enrichment of GO Biological process terms in the *CRG1* gene interaction set in normal growth conditions was calculated using Funspec [90].

**Analysis of Sterols Intermediates by Gas Liquid Chromatography - Mass Spectrometry**

Analysis of sterols was performed as previously described in Guan *et al.* [85] without modifications.

**Analysis of Lipid Droplets**

It was performed as described previously [91]. Stationary phase cells (42 hours) were treated with cantharidin (250 µM) for 2 hours. Cells were fixed with formaldehyde, washed and stained with the Nile Red solution (final concentration 0.4 µg/mL) for 10 min. Fluorescence excited at 488 nm and detected in the range from 550 – 575 nm.
